# Artifact Reduction in Simultaneous EEG-fMRI: A Systematic Review of Methods and Contemporary Usage

**DOI:** 10.3389/fneur.2021.622719

**Published:** 2021-03-11

**Authors:** Madeleine Bullock, Graeme D. Jackson, David F. Abbott

**Affiliations:** ^1^Florey Department of Neuroscience and Mental Health, The University of Melbourne, Melbourne, VIC, Australia; ^2^Florey Institute of Neuroscience and Mental Health, Melbourne, VIC, Australia; ^3^Department of Medicine (Austin Health), The University of Melbourne, Melbourne, VIC, Australia

**Keywords:** simultaneous EEG-fMRI, artifact, motion, ballistocardiogram, electroencephalography, BOLD

## Abstract

Simultaneous electroencephalography-functional MRI (EEG-fMRI) is a technique that combines temporal (largely from EEG) and spatial (largely from fMRI) indicators of brain dynamics. It is useful for understanding neuronal activity during many different event types, including spontaneous epileptic discharges, the activity of sleep stages, and activity evoked by external stimuli and decision-making tasks. However, EEG recorded during fMRI is subject to imaging, pulse, environment and motion artifact, causing noise many times greater than the neuronal signals of interest. Therefore, artifact removal methods are essential to ensure that artifacts are accurately removed, and EEG of interest is retained. This paper presents a systematic review of methods for artifact reduction in simultaneous EEG-fMRI from literature published since 1998, and an additional systematic review of EEG-fMRI studies published since 2016. The aim of the first review is to distill the literature into clear guidelines for use of simultaneous EEG-fMRI artifact reduction methods, and the aim of the second review is to determine the prevalence of artifact reduction method use in contemporary studies. We find that there are many published artifact reduction techniques available, including hardware, model based, and data-driven methods, but there are few studies published that adequately compare these methods. In contrast, recent EEG-fMRI studies show overwhelming use of just one or two artifact reduction methods based on literature published 15–20 years ago, with newer methods rarely gaining use outside the group that developed them. Surprisingly, almost 15% of EEG-fMRI studies published since 2016 fail to adequately describe the methods of artifact reduction utilized. We recommend minimum standards for reporting artifact reduction techniques in simultaneous EEG-fMRI studies and suggest that more needs to be done to make new artifact reduction techniques more accessible for the researchers and clinicians using simultaneous EEG-fMRI.

## 1. Introduction

Simultaneous electroencephalography (EEG) and functional MRI (fMRI) is a non-invasive imaging method first described over 25 years ago, and early on was mostly used for characterizing seizure location in epilepsy patients (1, 2). By combining the temporal resolution of scalp EEG with the spatial resolution of fMRI, it is possible to gain more information about brain activity than is possible with either technique alone (3). While simultaneous EEG-fMRI started out as a technique to locate epileptic activity, it is now used much more widely in neuroscience research (4), from studies looking at brain function in disease states such as schizophrenia (5, 6), to those investigating brain dynamics during behaviors such as decision making (7, 8) and sleep onset (9, 10).

**Figure 1:**
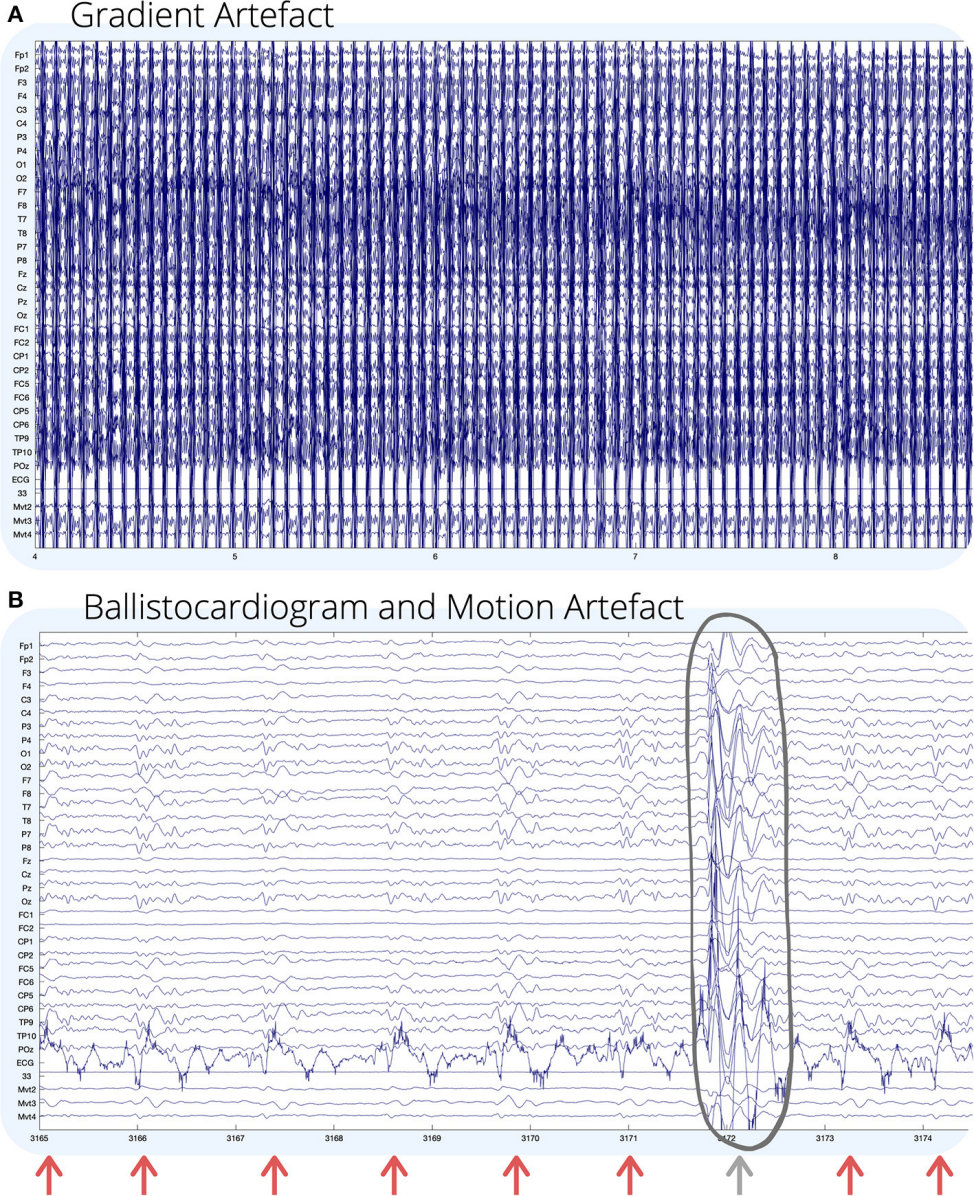
Visual examples of **(A)** gradient; and **(B)** ballistocardiogram (shown by arrows) and motion (circled) artifact on EEG recorded during fMRI. For **(B)**, GA has been removed using an adaptive average artifact subtraction (AAS) method (12). EEG channel numbers are given on the left of each figure. Channels below the ECG (mvmt 2–4) are recordings from carbon wire motion loops for measuring motion. The horizontal axis of each figure shows time in seconds. Environmental artifact is not seen visually in this recording, and for visual examples of environmental artifact, we refer the reader to (13, 14).

A technical challenge with recording simultaneous EEG-fMRI is that EEG recorded inside the MR environment is subject to large sources of noise, which can obscure neuronal activity, or induce artificial artifacts in the EEG recording (11). The four main sources of noise in EEG recorded during fMRI are outlined below, and some examples are shown visually in Figure 1.

- Gradient artifact (GA) is the largest source of noise in EEG-fMRI, and is used for fMRI acquisition due to the magnetic field gradients, which induce current in EEG electrodes up to 400 times larger than neural activity, therefore obscuring the EEG information of interest (15).
- Motion artifact occurs when movement of the subject's head within the scanner creates artifacts on the EEG due to induced current at electrodes when moved inside the magnetic field—occurrence explained by Faraday's law (16).
- Ballistocardiogram (BCG) artifact occurs due to the subject's cardio-respiratory patterns, specifically scalp pulse and cardiac-related motion, as well as the changes in magnetic properties of blood flow under the scalp (17).
- Environmental artifact occurring on the EEG recording is mostly due to interference from power line noise, ventilation, and lights in the MR room, as well as the vibration arising from the helium cooling pump used to ensure stability of the scanner's magnet (18).

Given the many artifacts and their impact, methods to reduce all artifacts are crucial to ensuring that the EEG recorded during EEG-fMRI is an accurate representation of brain activity. The earliest algorithm for artifact removal in EEG-fMRI was the average artifact subtraction (AAS) method, which was adapted to remove either BCG (19) or GA (15), and permitted the first fully simultaneous EEG-fMRI. However, due to temporal non-stationarities in template sampling, AAS-filtered EEG still contains residual artifact (15). In the years since the publication of the AAS method, many novel methods have been proposed to improve accuracy of EEG-fMRI, and research in this area is ongoing (20). An aim of this review is to distill the literature from these 21 years and provide updated recommendations about artifact reduction to those interested in practicing EEG-fMRI.

Previous published reviews, such as a recent paper from (20), have given a broad overview of the methods to reduce artifact in EEG-fMRI, but have not systematically reviewed all literature, nor given specific recommendations for researchers not familiar with this field. Perhaps the best example of practical recommendations is a visual article by Mullinger et al. (21), which shows step-by-step optimization for specific EEG-fMRI equipment. While these reviews are useful, there remains a gap: a systematic review of artifact reduction methods in EEG recorded during EEG-fMRI is provided to underpin a set of clear recommendations that make it easier for researchers to make informed decisions about reducing artifact in any EEG-fMRI study.

We aim to:

1. Systematically review EEG-fMRI artifact reduction methods and provide some clarity regarding a potential “gold standard” approach;
2. Systematically review all papers published in the last 4 years that have used EEG-fMRI to determine the artifact reduction techniques used in contemporary practice.

The results of our review lead to guidelines for EEG-fMRI usage, and recommendations to help ensure best practice techniques are widely used.

## 2. Methods

### 2.1. EEG-fMRI Artifact Reduction Techniques

The objective of the first literature search was to identify novel artifact reduction techniques for EEG-fMRI from literature published since the first EEG-fMRI artifact reduction paper appeared in 1998. This review has a specific focus on EEG-informed fMRI, where results of EEG are used to inform the fMRI analysis. Web of Science database was searched for papers related to artifact reduction in EEG-fMRI, in English, from years 1998 to 2019 (January 17, 2020). Document types were categorized into article, proceedings paper, review, data paper, early access, or book chapter (exclusion of editorial material, meeting abstracts, and corrections). The search terms are outlined as follows:

(TS = (((eeg OR electroencephalography) AND (“functional mri” OR fmri OR “functional magnetic resonance imaging”)) AND (artifact OR artifact* red* OR filter* OR denois* OR classif*))) AND LANGUAGE: (English)

The title, abstract, and, if required, methods sections of resultant papers were then manually interrogated to include only literature which fitted the following criteria: data from *human* subjects, recorded using *EEG and fMRI*, where EEG and fMRI are recorded *simultaneously*, i.e., EEG recorded inside the MR environment, while fMRI scanning is occurring.

The final step was to determine which of the resultant papers outlined *novel artifact reduction* techniques for the EEG data recorded during EEG-fMRI. Artifact was defined as any source of activity, which appears in the EEG dataset, but is not neuronal in origin. Artifact reduction can include (1) methods to reduce the raw recording of artifact during EEG-fMRI acquisition, and (2) methods to filter out artifact during the post-processing of EEG. A method was considered novel if there were no previous publications outlining its use for reducing *any* artifact on EEG data recorded during EEG-fMRI. By this definition, papers that outline the use of an existing published method, but implemented to reduce another type of artifact, are not considered novel. Although this review focuses on methods that improve EEG clarity, it is accepted that some techniques that improve EEG quality may also impact fMRI quality, positively or negatively. Other literature has covered the impact of the EEG system on artifact in the fMRI recording: for most commercial EEG-fMRI systems, artifact from EEG is considered far lower than that generated by fMRI (11, 22–24).

### 2.2. Contemporary Use of Artifact Reduction Techniques in EEG-fMRI Studies

The primary aim of the second review was to acquire information about the setup and post-processing methods employed by researchers for reducing artifact in contemporary EEG-fMRI studies (2016–2019) (Aim 1). A secondary aim was to understand the level of diversity in recently published studies using EEG-fMRI (Aim 2). Given these aims, there were three primary areas of interest for the included studies: (1) Methods of EEG-fMRI setup (pre-recording) for artifact reduction (Aim 1); (2) post-processing (post-recording) methods for artifact reduction (Aim 1); and (3) measure of interest on EEG used to drive the EEG-fMRI analysis (Aim 2).

The literature search covered the databases—Web of Science, PubMed, and Scopus—and included all articles between January 1, 2016 and December 31, 2019, with the following terms: EEG-fMRI, ERP-fMRI, EEG-BOLD, and their derivatives, including long hand spelling, and words in alternate orders. Key search fields were as follows: Title, Abstract, and Keywords. The inclusion criteria for EEG-fMRI studies is fully simultaneous EEG-fMRI in human population. The definition of simultaneous EEG-fMRI and human population is the same as outlined in part 1, with EEG-fMRI studies using interleaved scanning not considered fully simultaneous. Studies included in this review must have a full methods section, and be either English language publications, or have a translation readily available.

## 3. Search Results

### 3.1. EEG-fMRI Artifact Reduction Techniques

**Figure 2:**
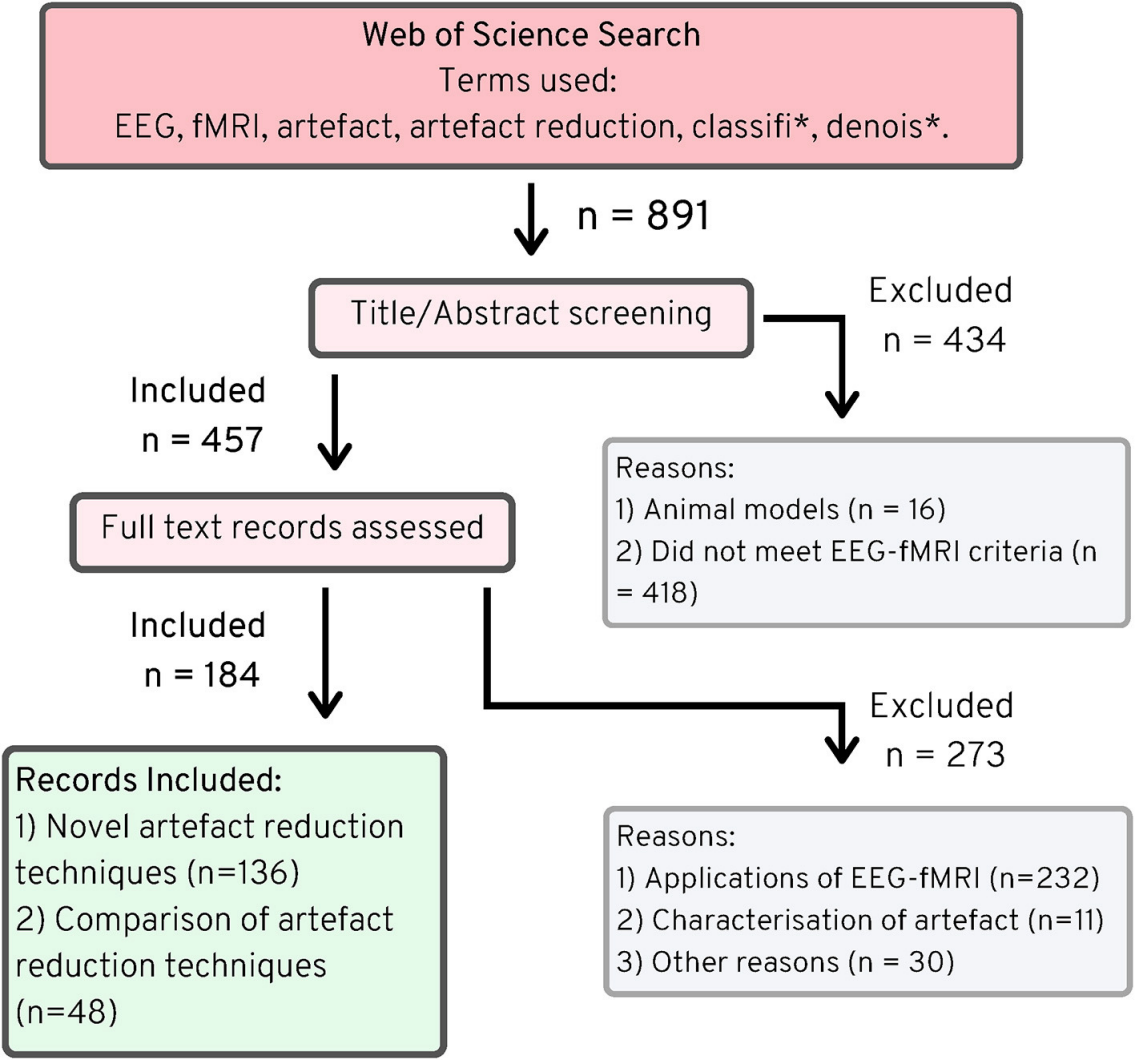
PRISMA chart (25), EEG-fMRI novel artifact reduction methods, 1998–2019.

The Web of Science search returned 894 articles (891 unique), of which 457 fitted the criteria of simultaneous EEG-fMRI as outlined in the methods, and of those, 136 described novel artifact reduction methods (Figure 2). Many of the papers that did not meet the criteria for simultaneous EEG-fMRI described either only EEG *or* only fMRI data. Some papers described data from both modalities, but these data were not simultaneously acquired. Sixteen papers detailed imaging in animal models, without any human imaging. When assessing whether the remaining papers described novel artifact reduction techniques for *EEG* data, 273 were excluded, with the most common reasons being:

1. Applications of EEG-fMRI for answering a clinical, neuroscience, or behavioral question, with no novel artifact reduction technique shown;
2. Characterization of EEG artifact during EEG-fMRI, but no method to remove it;
3. Other reasons, such as:
  - EEG-fMRI review articles;
  - Methods for EEG-fMRI data fusion;
  - Methods for artifact reduction in *fMRI* data only;
  - Proposals of methods for improving EEG-fMRI acquisition, but with no data validation.

The search also identified 48 papers that described comparisons of EEG artifact reduction methods for EEG-fMRI, without proposing any new method. While, these papers do not describe novel methods, they were used to inform the discussion in the following sections in order to provide an objective measure of best practice in EEG-fMRI artifact reduction.

### 3.2. Contemporary Use of Artifact Reduction Techniques in EEG-fMRI Studies

**Figure 3:**
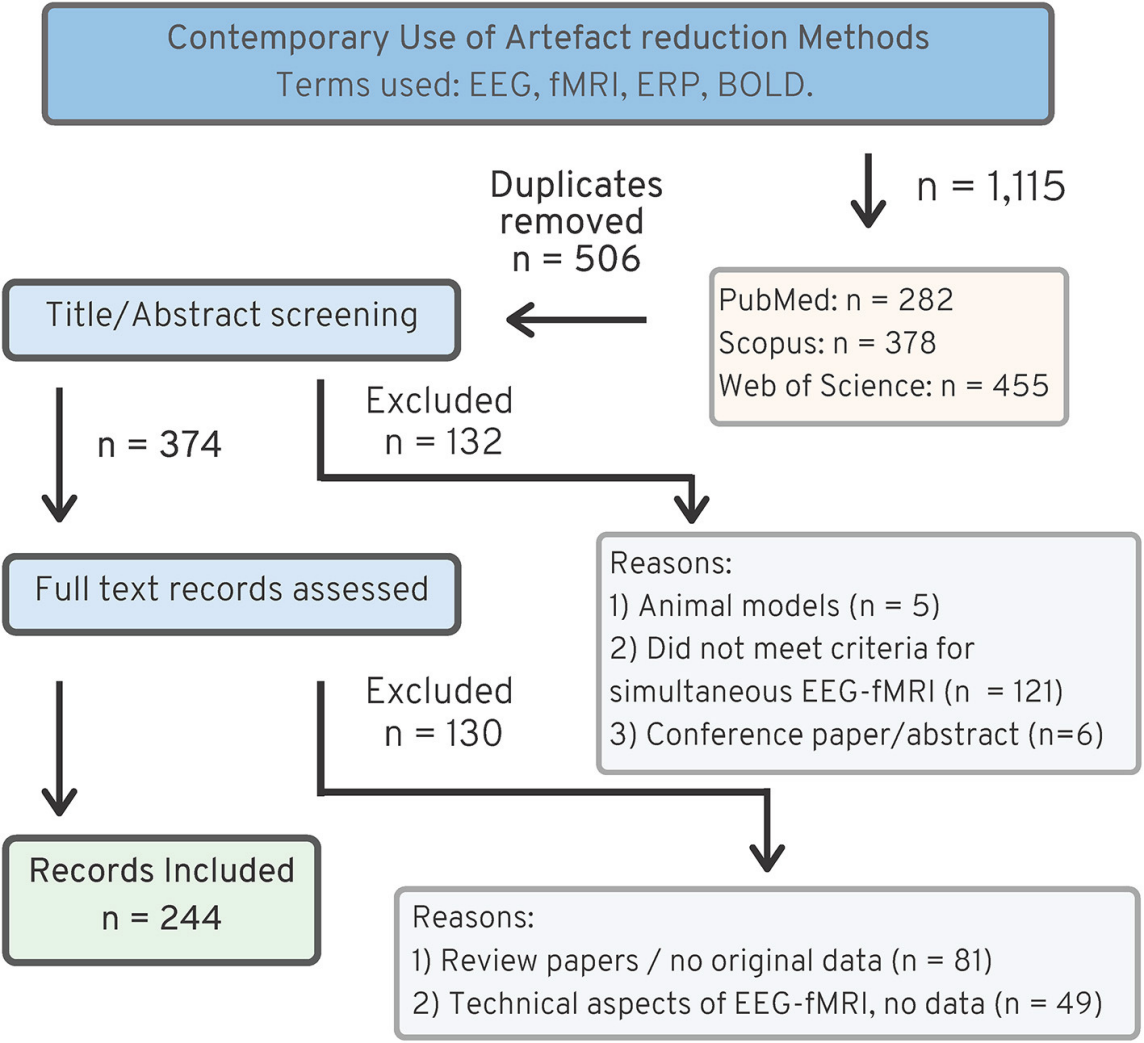
PRISMA chart (25), showing search strategy for recent EEG-fMRI papers, 2016–2019.

Figure 3 shows the search strategy used for finding literature relating to artifact use in contemporary EEG-fMRI studies. The original search returned 506 original articles, after excluding duplicates from each of the databases. Screening of papers by title and abstract excluded 132 papers, and full text screening of papers excluded another 130 papers. The most common reasons for exclusions were as follows: animal studies (*n* = 5), review papers with no original data (*n* = 81), papers where data are not from simultaneous EEG-fMRI (*n* = 121), and papers discussing technical aspects of EEG-fMRI without original data in human subjects (*n* = 49). After exclusion, the review includes 244 EEG-fMRI papers for analysis.

## 4. Artifact Reduction: Recommendations and Contemporary Use

This section is divided into subsections based on the four main EEG-fMRI artifact types [GA, BCG artifact, motion artifact, and environmental artifact] and includes results from both reviews in each section. For each artifact type, recommendations are made for the best practice for reduction of that artifact, followed by contemporary usage statistics to contrast best practice vs. current practice. In the interests of space, not all 130 novel artifact reduction techniques revealed in the literature search are covered in detail in the following discussion. A comprehensive list of all techniques is provided in additional tables in the Supplementary Material.

A final subsection details the software toolboxes used to reduce artifact in contemporary studies. Although we do not evaluate software toolboxes in this review, understanding the most commonly used software could help to identify the reasons researchers are using a particular artifact reduction technique in contemporary studies.

### 4.1. Gradient Artifact Reduction

GA, or imaging artifact, is the largest artifact seen on EEG recorded during fMRI, and occurs due to the strong magnetic field changes during fMRI scanning. GA amplitude can be over 400 times larger than the EEG of interest (15), making its removal crucial for a successful EEG-fMRI study. Early EEG-fMRI studies avoided GA by altering the time of scanning, so that fMRI was acquired either in response to an EEG event such as an epileptic spike (2), or by interleaving EEG and fMRI acquisition (26). However, alternating fMRI and EEG acquisition cannot be considered simultaneous, and useful neuronal information is lost when data are acquired in this way. In order to be simultaneous but still to avoid GA, a method known as Stepping Stone Sampling (27) was developed, which avoids GA by altering the fMRI pulse sequence so that EEG can be sampled during times when GA is known to be negligible. However, Stepping Stone Sampling has been largely superseded because newer post-processing methods allow for equivalent or better (28) recovery of neuronal activity from continuous scanning using conventional fMRI pulse sequences.

#### 4.1.1. EEG-fMRI Setup

If not avoiding GA using Stepping Stone Sampling or another method, the EEG equipment must have sufficient dynamic range to record both the GA and the underlying EEG to facilitate subsequent post-processing to separate these signals. Newer amplifiers that are wireless and can adapt amplification depending on MR gradient sequences are in development (29) but are not currently available. One problem with recording EEG in the MR environment is that EEG leads can form large loops, which increase the prominence of induced current from GA, and may be dangerous if heating occurs (30). To reduce the size of EEG cable loops in the MR environment and to reduce the magnitude of GA being recorded, EEG cables should be twisted (22, 30–32), as short as possible (32, 33), and located centrally on the EEG cap (34). Re-wiring the EEG cables based on scanner specific artifact recordings may further reduce the amount of GA recorded in the EEG (35). Also by optimizing the setup of EEG equipment, positioning the subject with nasian 4 cm posterior to isocenter (36) may reduce GA contamination of EEG.

#### 4.1.2. Post-processing

Template methods, such as AAS can be used to remove GA, and blind source separation (BSS) methods, as well as other filtering methods, have been proposed as alternatives.

##### 4.1.2.1. Template Methods

Template methods are techniques that create an estimate of the artifact, which is removed from the noisy data, to obtain clean EEG. GA is assumed to be repetitive and time-locked to each repetition time (TR) during fMRI scanning, whereas the neuronal EEG of interest is assumed to be random and fluctuating without regard to TR. Template methods average the signal over many TRs, leveraging the fact that the neuronal EEG signal, which is not time locked to the TR, will average out toward zero, leaving the GA signal, which can be subtracted from the recording to give the cleaned EEG (37). Providing that the assumption of GA stability and EEG fluctuation, with regard to TR timing, throughout the recording is valid, template methods can provide a relatively easy and reliable way to clean the EEG.

Temporal jitter of EEG and MRI scanner clocks leads to slight differences in the calculation of templates, resulting in residual GA contamination of EEG after artifact removal by template methods. Therefore, to accurately sample the GA and use template methods most effectively, MR and EEG clocks should be synchronized (27, 38–40), preferably using a synchronization hardware device (38). For studies where synchronization hardware is not available, post-processing methods, such as interpolation (41), auto-correlation (42), time continuous cubic spline model (43), or least across squares variance (44), can be used to realign EEG and fMRI data. However, the use of synchronization hardware, available with many commercial EEG-fMRI systems, remains the optimal approach.

The template methods for GA removal described in the literature differ from each other by the way that the template is generated, as well as how the template is subtracted from the recording. The earliest template method, AAS, considers GA at each TR epoch in the temporal domain, and applies a moving average filter (over some number of immediately preceding TR epochs) to remove GA, leaving the cleaned EEG data (15). Other template methods, such as template sets based on cubic spline interpolation (45) and hierarchical clustering (46) use templates derived from the whole recording rather than temporally preceding, and filter data based on these clusters, or sets, of templates.

There have been few comparisons of template methods available in the literature. However, even in the original paper that describes it, cubic spline interpolation did not outperform AAS, unless additional motion parameters from an MR compatible camera (or, it was suggested head displacement parameters from fMRI analysis could possibly be substituted) were also incorporated into the algorithm (45). The addition of head displacement parameters gives an additional information to the cubic spline model, and it is unclear if using AAS with this additional information would have produced comparable results. Hierarchical clustering showed improved performance compared with a pipeline of AAS and interpolation of EEG and MR data, but was not implemented on a dataset where EEG and MR timing were synchronized (46). It is therefore questionable whether hierarchical clustering could improve the performance of AAS if adequate synchronization was employed, and it is difficult to suggest this method without appropriate independent validation. The literature search showed that of the three template methods described above, only AAS has been independently scrutinized, and many papers have built on or suggested additional factors for its implementation.

AAS, when used for removing GA, can be prone to residual artifact due to fluctuations in GA over the course of the recording, such as from head motion (47), drift in EEG sampling rates (12, 38), or from contamination due to BCG artifact (48). Methods to reduce the resultant residual artifact present after AAS include using extra steps such as pre-processing template data (49), or improving the template estimation by weighting templates based on proximity (50), linear regression (51), cross-correlation (52), or adaptive filtering (53). However, these methods that describe extra steps for removing residual artifact with AAS are dealing with the residual artifact from non-synchronization of clocks, and if they compare their method with AAS, it is AAS without synchronization (49, 51, 53). One paper [cross-correlation (52)] considered how synchronization of EEG and MRI would affect their method, and stated that their method would likely provide a similar magnitude of GA reduction compared to using AAS with synchronization. Weighting templates based on proximity (50) was tested using synchronized EEG and MRI data, but the protocol for MR acquisition was Stepping Stone Sampling (27), an uncommon MRI sequence. The paper was aimed toward retaining high-frequency signatures of EEG, rather than reducing GA (50), and therefore, the findings of this paper will be more relevant to those working in the field of high-frequency EEG.

The biggest issue with using AAS to remove GA is that BCG and motion artifact affect the accurate calculation of AAS templates (47), with these inaccurate templates often causing the residual artifact seen in recordings (48). In 2009, two groups independently published methods showing that motion information, obtained from fMRI head displacement parameters, can be added to the calculation of AAS templates to improve GA removal (12, 54). The addition of head displacement parameters to AAS is particularly suited to cohorts such as children where motion is unavoidable, and is particularly beneficial when head displacement during the time of the scan exceeds 1 mm. The addition of motion parameters to AAS showed an experimental increase event-related potential (ERP) power of 10–40%, (12), as well as a reduction in residual GA of 20–50% (54). Recently, it has been suggested that information from hardware that directly records head motion, rather than fMRI head motion parameters, could also be used to improve the calculation of AAS templates (55, 56). A discussion of direct motion hardware is given later in section 4.2.3. Finally, a recent study capitalized on the fact that the GA is stable, and using a model of the GA and the principles of Faraday's law enable us to determine which volumes were affected by head motion, and thus adjust the AAS templates accordingly (47).

##### 4.1.2.2. Blind Source Separation Methods

BSS techniques are a group of data-driven methods that aim to decompose a mixed signal (input signal) into its multiple original sources (57). In EEG-fMRI, the noisy signal (EEG and artifact) is the input, with output components ideally being separated into components representing neuronal signal or sources of noise. BSS typically uses statistical properties, such as Gaussianity, variance, or correlation to identify these sources, even when no prior information is given (58). As such, BSS methods can be preferable to template methods when information about the signal, such as MR slice timing, is unknown.

Popular BSS methods employed to reduce GA in EEG-fMRI studies include independent components analysis (ICA) (59), principal components analysis (PCA) (60), canonical correlation analysis (CCA) (61), and blind source extraction (BSE) (62). Variations of these main methods for GA reduction include the optimal basis set (OBS) method (63), which uses template methods alongside PCA, independent vector analysis (IVA) (64), which employs ICA over multiple EEG channels, and single value decomposition (SVD) (65), a subset of PCA.

Of the BSS methods described, ICA and OBS have been independently compared for removal of GA from EEG-fMRI data, with ICA showing good results in simulated data, but OBS outperforming ICA in experimental data (66). A difficulty with using BSS methods is the challenge of correctly choosing the components that represent artifact and those which represent neuronal activity, especially if no prior knowledge about the signal is available. There is a chance that by rejecting too many components, neuronal signal, as well as artifact, is removed from the recording and important information is lost (67).

##### 4.1.2.3. Other Methods

*4.1.2.3.1. Frequency Filtering*. Frequency filtering uses algorithms such as the Fourier transform (FT) to represent the EEG signal in the frequency domain, where frequencies corresponding to GA can be removed (26). However, given that some GA occurs at frequencies corresponding to neural activity, frequency filtering is not suitable for accurately reducing GA without losing important neuronal information (26). Frequency filtering using the FT has been suggested as an option for real-time removal of GA, where computational speed of the algorithm is more important than a potential loss of neuronal signal (68). The Taylor–Fourier transform expands the FT by allowing for fluctuations in harmonics during each temporal segment of data, regardless of how small, (69), allowing it to capture amplitude and phase variations that would not be possible with the FT alone. For EEG-fMRI, the Taylor–Fourier transform method creates a dynamic template at each individual TR, and was shown to improve removal of residual artifact, but was not validated against AAS or any other GA removal technique (70).

*4.1.2.3.2. Dictionary Learning*. Another approach to GA reduction, dictionary learning, is a computationally expensive way of separating the GA and EEG, which has shown an ability to remove GA from contaminated EEG, but has not been tested against other commonly used GA removal methods, either in the original paper or independently (71). Therefore, we cannot recommend the dictionary learning model for removing GA until further validation of the method is available.

##### 4.1.2.4. Comparison of Methods

Few papers independently compare GA removal methods for post-processing of EEG-fMRI data. In 2007, Grouiller et al. (66) published a study that compared AAS, OBS, ICA, and FT in both simulated and experimental EEG-fMRI data. Results showed that while ICA performed well for simulated data under 30 Hz, AAS and OBS performed better in experimental data, and FT had the poorest results. Other studies have compared AAS and OBS only, especially in their commonly used software, with AAS often being implemented from commercial software Brain Vision Analyser, and OBS implemented by an open source plug in through EEGLAB (63). Comparisons of AAS and OBS suggest that OBS may be more adept at removing artifact (67, 72), although it has been suggested that the increase in artifact removal seen from OBS is due to additional BCG removal as well as GA removal, and which may have been removed during other post-processing steps for removing BCG later (72, 73). Overall, the methods of AAS and OBS appear similar for cleaning of GA from EEG-fMRI, provided that adequate synchronization of MR and EEG clocks occur. OBS may clean additional BCG from the data during the GA cleaning step, and therefore should conservative artifact removal be required, AAS with synchronization may be preferred. Future studies that independently compare some of the less known methods, such as dictionary learning, and template methods (not AAS), would help to improve guidance for GA removal from EEG-fMRI studies.

#### 4.1.3. Recommendations for Removing Gradient Artifact

**Recommendations for Removing Gradient Artifact from EEG-fMRI:**

- EEG cable length (from cap to amplifier) as short as possible.
- EEG cables (cap and cable bundle to amplifier) twisted to reduce loop areas, minimizing magnetic field effects.
- EEG cable position central to the bore and patient cap.
- Patient nasian 4 cm from isocenter.
- Synchronization of EEG and MR clocks, preferably using hardware rather than interpolation methods.
- Post-processing removal of GA with either AAS (conservative, possible residual artifact) or OBS (rigorous, possible loss of neuronal information).
  - Additions to these algorithms (e.g., including head motion parameters) may remove GA more accurately.

#### 4.1.4. Contemporary Use of GA Removal Methods

**Figure 4:**
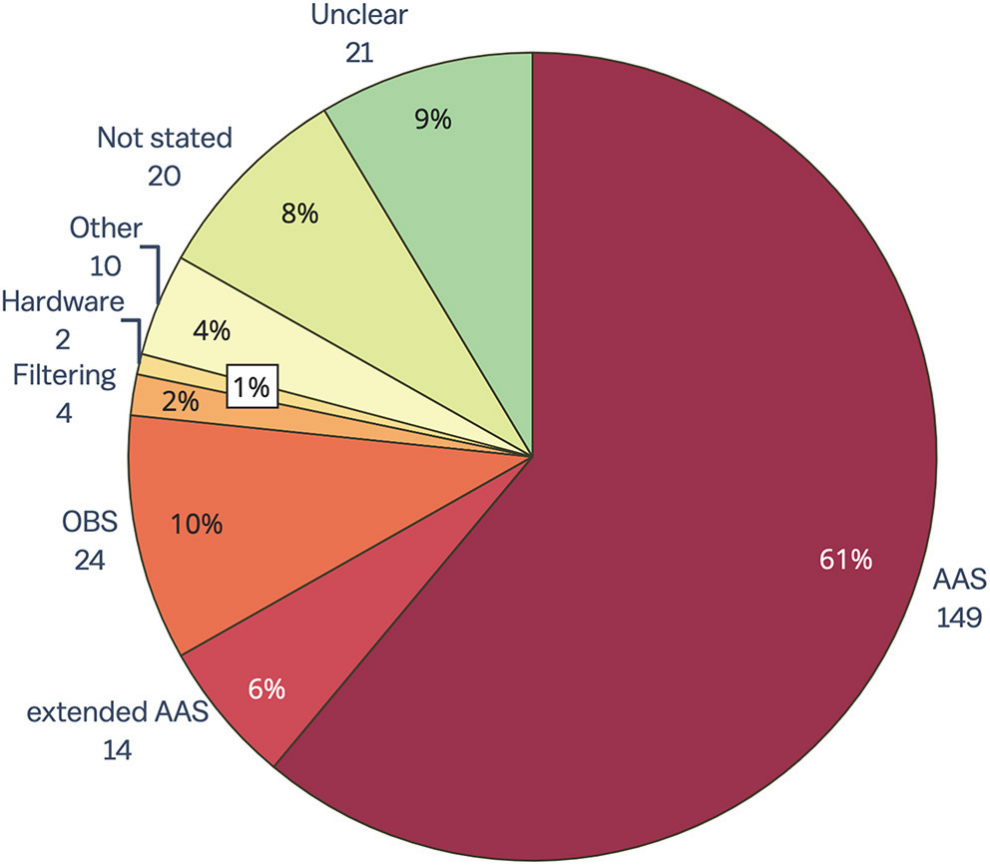
Gradient artifact removal in EEG-fMRI papers, published between 2016 and 2019 (*n* = 244). AAS, average artifact subtraction; OBS, optimal basis set.

The results of the review of contemporary EEG-fMRI studies show that AAS (15) is still overwhelmingly the most widely used technique for removing GA from EEG acquired during fMRI studies (61%, Figure 4). The OBS method [48] is used in only 10% of studies, while filtering, hardware and all other methods make up only 7% of GA removal methods. Despite there being a number of published papers outlining extensions to the AAS method to make it more accurate (for a full list, see the Supplementary Material), 6% of papers published between 2016 and 2019 report using an extended AAS method. In 17% of papers, the GA removal method was either unclear (9%), or there was no indication of GA removal at all (8%). GA removal method was considered “unclear” if the paper mentioned removing GA but did not state a specific method, or the paper commented on the commercial software used to remove artifact but did not outline method or steps taken within that software.

A surprising result from this review is that despite the many reported benefits of synchronizing EEG and MR clocks for later artifact removal (38, 39), less than 50% of the EEG-fMRI papers included in this review reported using synchronization hardware (for full data, see the Supplementary Material). The low percentage of synchronization hardware use may be partially due to the inadequate reporting of methods in the literature. Regardless, the low number of papers reporting use of synchronization is concerning given that effective GA removal using template methods such as AAS (the most commonly used method) relies heavily on adequate synchronization (38). We recommend that synchronization hardware be used in all EEG-fMRI studies so that GA can be accurately removed from EEG, and also recommend that authors include synchronization use in their methodology when publishing research.

### 4.2. Ballistocardiogram Artifact

The BCG, pulse, or cardio-ballistic artifact, refers to contamination of the EEG due to pulsatile motion associated with cardiac output (65). BCG voltage has been measured at around 50 μV, which is in the physiological range of typical EEG signals of interest (19), and therefore is a considerable confound that cannot easily be avoided. A technique known as pulse triggered scanning (74) has been proposed as a way to avoid the main source of BCG artifact, but is only practical for task-based fMRI and requires specific technical expertise to set up. Therefore, most studies opt to use standard fMRI sequences, and rely on accurate recording of pulse and physiological signals during EEG-fMRI to estimate and remove BCG artifact during post-processing of EEG data.

#### 4.2.1. Measurement of BCG

Monitoring physiological signals such as heart and breathing rate can give a baseline estimate of BCG artifact and enable better removal during post-processing of EEG. Common methods to measure physiological signals include using an additional electrode to measure ECG (19, 30), as well as the use of photoplethysmography (PPG) or a respiratory band, which is sometimes included with the MRI scanner setup (39). Choice of measurement may depend on the post-processing methods chosen, and the researcher needs to ensure that any additional signals measured can be accurately synchronized with EEG and MR timings.

#### 4.2.2. Post-processing

From a post-processing perspective, BCG artifact removal is arguably more difficult than GA removal because BCG varies throughout the recording (46, 75, 76). BCG artifact differs across scalp electrodes, and may be contributed to by pulsation motion of the scalp, electromagnetic effects of blood flow under the scalp (Hall voltage), and movement related to respiration (17). BCG can be removed with various post-processing methods including template methods and BSS methods. Many of the methods outlined for GA removal in the previous section (section 4.1.2) have been adapted for BCG removal.

##### 4.2.2.1. Template Methods

Template methods can be used to remove BCG artifact from EEG-fMRI data in a similar way to GA methods described in section 4.1.2. However, unlike for GA, where the beginning of each template is defined by MR trigger events at the beginning of each TR, the beginning of each BCG template is usually determined based on the peaks seen during simultaneous recording of physiological signals, such as ECG, alongside EEG-fMRI.

BCG removal from EEG recorded inside the MR environment was first proposed by Allen et al. (19) in 1998, using the AAS template method with QRS complexes from ECG used to mark the beginning of each BCG template. An issue with using AAS method for removing BCG artifact is that it assumes that all BCG will be of the same or similar shape, and only slowly vary across the whole recording, whereas evidence from literature has shown that BCG is highly variable across the recording and has many different shapes (46, 77). Because of this problem, adaptations of AAS for BCG removal have been proposed, including using exponential weights for previous BCG instances to account for the variability of BCG artifact (30), data warping the AAS template to account for shape differences (77), as well as additional steps such as wavelet decomposition to help remove residual BCG artifact left by inaccurate BCG templates (78). These methods, while shown to improve the correlation of EEG from outside scanner with filtered inside scanner recordings compared to AAS alone (77, 78), still do not account for all the dynamic changes that occur in BCG between subsequent heart beats. The dynamic time warping approach goes some way to account for shape changes that occur between BCG instances, but still uses previous iterations of BCG to determine the general shape of the waveform (77).

One of the problems associated with AAS is that BCG artifact can continue longer than a single heartbeat event, and therefore might overlap with the next BCG event in the recording. In fact, a study showed that when recorded at 3T, BCG artifact that overlapped into the next BCG template accounted for up to 30% of all recorded BCG (46). Several methods to overcome this overlapping of BCG artifacts has been proposed: the moving General Linear Model (mGLM) (79) and hierarchical clustering (46). The mGLM method uses a Fourier series to model each BCG template, and therefore does not cut off the template at the beginning of the following heartbeat. The mGLM model is similar to AAS in that the BCG template is updated temporally and relies on information from previous templates in its generation. In fact, if there is no overlapping of BCG, then mGLM will act identically to AAS (79). Clustering algorithms, such as hierarchical clustering (41, 46) and k-means clustering (80), account for overlapping BCG components by dividing all BCG instances from the entire recording into groups of similar templates, which can then be removed. Hierarchical clustering was tested on EEG-fMRI data from a sample of 29 healthy people at 1.5 T, as well as 15 epilepsy patients and one healthy person at 3 T. Results showed that BCG template duration was longer for higher magnetic field MRI (3 T) compared with 1.5 T recordings, and that the BCG templates not only vary between subjects, but within the recording, with clusters not ordered throughout the recording (46). This implies that techniques such as AAS-based methods and even mGLM may work sub-optimally, due to relying on information from previous BCG for determining the shape of the BCG template. By using the whole recording and clustering all BCG instances, the hierarchical clustering method is able to overcome the limitations of overlapping BCG as well as the variability of BCG. When compared to AAS and OBS, k-means clustering has showed slightly less BCG attenuation, but better neuronal preservation, compared with OBS—and vice versa compared with AAS—during an EEG-fMRI visual task at 7 T (80). Therefore, it has been suggested that clustering algorithms may be a good trade off for both attenuating BCG and preserving neuronal activity, compared with AAS and OBS (80).

Template methods for BCG removal typically require an accurate recording of physiological signals, particularly QRS complexes from ECG, that are used to generate BCG templates. While the AAS method uses amplitude information to detect the R peak of the QRS complex, other algorithms have been described for detecting R peaks. These algorithms include a modified Pan–Tompkins algorithm, which uses width and slope measurements as well as amplitude to detect R peaks (81), and k-Teager Energy Operator algorithm, which determines the total energy of the signal in a particular frequency range to identify R peaks (63, 78, 82, 83). Other methods for detecting R peaks have also been suggested, such as using the amplitude of the difference between the positive R peak and negative peak between S and T waves to determine the location of the R peak, as well as using a window of intervals based on expected heart rates to determine approximate location of peaks (84). We did not find any direct comparisons of the methods for R peak detection for BCG artifact recorded during EEG-fMRI. The most commonly cited R peak detector, k-Teager Energy Operator, was proposed for the specific purpose of finding peaks for BCG that occurs during EEG-fMRI (78), and therefore may be a better fit than older algorithms such as Pan–Thompkins, which was initially developed with the purpose of identifying peaks during ambulatory ECG (85). A potential issue with using the k-Teager Energy Operator is identifying the best value of k (the expected frequency of peaks). The default value, which is calculated based on the sampling rate of data, may identify too many false positive peaks (83), so heuristic customization may be required. An incentive for using k-Teager Energy Operator is that it can be used with other physiological recordings of BCG, such as from electrooculogram (EOG) electrodes attached to the side of the head, rather than using an ECG electrode (78). In this way, motion aspects of BCG may be better represented in EOG electrodes, without requiring direct motion recording from additional hardware, and without lag between QRS complexes recorded in ECG leads and BCG artifact seen on the head.

##### 4.2.2.2. Blind Source Separation Methods

Blind source separation techniques, ICA, PCA, CCA, as well as OBS, have also been applied to removing BCG artifact from EEG-fMRI (for all methods, see Supplementary Material). BSS techniques offer a data-driven way to remove BCG, regardless of the variability of the BCG artifact, and without the necessity of measuring physiological signals.

*4.2.2.2.1. Principal Components Analysis*. PCA was first described alongside ICA, for BCG removal by (86). While, PCA was shown to be capable of removing BCG and improving the detection of epileptic spikes, it was concluded that ICA performed better (86). Since then, research into blind source separation methods for BCG removal in EEG-fMRI has focused on ICA rather than PCA, unless some extension or modification of the PCA algorithm was used.

One such algorithm that builds on PCA is the maximum noise fraction, which uses similar processes to PCA, but differs in that it derives two matrices—one for the signal (which is assumed to have a degree of smoothness) and one for estimated noise (which is assumed orthogonal to the signal). For the purpose of separating BCG from neuronal electrical activity, the BCG is assumed the temporally smoother “signal” and neuronal activity the “noise.” The algorithm generates output components based on maximizing signal to noise ratio (SNR) between these matrices (87). In this way, the maximum noise fraction approach can be considered superior to ICA or PCA, because the components generated from the maximum noise fraction are automatically sorted into BCG and likely neuronal activity components (87). In a test of four subjects, with BCG removal compared between ICA and maximum noise fraction, the maximum noise fraction components chosen to represent BCG were more specific to the actual BCG artifact in comparison with the independent components from ICA (87).

Another method that builds on PCA is to precede it with empirical mode decomposition (EMD) (88, 89). Empirical mode decomposition first decomposes the signal into intrinsic mode functions with various frequencies and amplitudes, and then PCA is undertaken on each of the intrinsic mode functions (89). The combination of EMD and PCA is more sensitive to temporal variation that occurs in BCG, compared to application of PCA or ICA alone (89). EMD-PCA has been tested for many different EEG-fMRI paradigms, and has shown better removal of BCG from ERP task EEG-fMRI compared with OBS and AAS (88–90). In resting state EEG-fMRI, EMD-PCA removal of BCG improved the detection of most parameters measured from the EEG and was shown to be a useful technique when a reference signal such as ECG is not available to estimate BCG onset (91).

*4.2.2.2.2. Optimal Basis Set*. The OBS method (63), section 4.1.2, uses PCA in conjunction with a template method to remove artifact. Like AAS, OBS for BCG removal relies on accurate generation of templates and can benefit from techniques to precisely identify QRS complexes from physiological data. Adaptations of OBS include real-time monitoring of EEG data during fMRI (92) and correlation between PCA components and ECG signal, which helps to reduce the issues with temporal variation of BCG (93). The original OBS method used three main principal components (PCs) to represent the BCG from a recording. However, independent studies have shown conflicting data, with some suggesting that altering PC number for each individual dataset alters the outcome (73), whilst others show that OBS is relatively stable regardless of PC number chosen (76). A study using high-field 9.4 T MRI showed that OBS was not successful in extracting ERPs, unless ICA, with specific methods for choosing ICs, was used alongside it (94). Therefore, the evidence suggests that for removing BCG artifact, OBS is best used in conjunction with ICA, provided that IC selection is carefully monitored.

*4.2.2.2.3. Independent Components Analysis*. The main advantage of ICA, compared with template methods, is the ability to combine spatial and temporal information about BCG, without the need to record physiological signals, in a process which is fully data driven (95). However, there is a risk one may remove independent components (ICs), which relate to neuronal activity rather than artifact. Common methods for sorting BCG ICs include correlation (95, 96) variance (97), auto-correlation, frequency sorting, or peak to peak measures, all of which rely on the ECG channel as a reference [for a full overview, see review in (76)]. More recent methods of sorting BCG ICs include using Mutual Information algorithm (65, 98), clustering the results of multiple ICA runs (99), using a Magnitude Squared Coherence Function (100), and finally using a projection of QRS complexes from ECG into the IC space, followed by k-means clustering of all ICs (101). In addition, a variation of ICA, constrained ICA (cICA), may improve on ICA alone by using a prior estimation of BCG artifact to help derive ICs which are likely to closely match the BCG artifact (102–105). A comparison study of common IC sorting methods showed that peak to peak sorting of BCG ICs outperformed sorting by variance measures (76). However, this is in contrast to a second comparison study that showed that depending on the weight given to artifact removal or neuronal activity retention, the effectiveness of each of the IC sorting method differed (101). In this second study, sorting ICs by frequency showed the best overall reduction of BCG regardless of neuronal activity loss, while sorting ICs using auto-correlation showed the best outcomes for preserving neuronal activity and removing BCG, tested on 7 T data (101). When taken together, these results suggest that sorting of ICs is difficult, and may be confounded by scanner setup, magnetic field, as well as the study requirements that prioritize either full BCG removal, or neuronal activity preservation.

*4.2.2.2.4. Canonical Correlation Analysis*. CCA is a technique that takes two sets of basis vectors and computes linear sets of vectors from each set (106). CCA was adapted for removing BCG from EEG-fMRI by temporally segmenting the data based on BCG artifact timing, followed by employing CCA on consecutive epochs of BCG instances, to determine the underlying sources responsible for common signal between the segmented epochs (106). It was argued that CCA would be superior to ICA or PCA, as it is able to take into account temporal (periodicity) and spatial (topography) aspects of the BCG due to taking consecutive BCG instances into account (106). Originally requiring manual identification of BCG artifacts on EEG (106), later implementations of CCA used ECG and R peak detection for determining BCG onset (107). CCA performance has been observed to be better than AAS for some subjects, whereas for others the performance can be quite similar to AAS (108). In ERP studies, CCA appears to perform similarly to OBS or AAS when trial number is high, and better when trial number is relatively low (107). Results of these studies suggest that CCA may be a useful post-processing technique for studies where BCG variability within a subject is high, and recording time of the EEG-fMRI study is short.

##### 4.2.2.3. Other Methods

Other methods for BCG removal include techniques that filter BCG based on some of its characteristics such as its spatial (109), spectral (110, 111), or morphological (112, 113) features. In addition, several papers outline the application of advanced filters such as Kalman filtering (114, 115), for BCG removal during EEG-fMRI. Finally, Abolghasemi and Ferdowsi (116) used dictionary learning for removal of BCG from EEG data, where a sparse dictionary is able to model the BCG data from EEG-fMRI data.

Removal of BCG by its spatial characteristics (109) was shown to be adequate compared with AAS, but only improved on AAS in the instance when BCG effects were temporally and spatially locked with EEG events of interest. Source Extraction (CSE), based on the spectral components of BCG, has been shown to be able to remove BCG from signals and result in good SNR and visual evoked potential (VEP) recovery, in comparison to AAS and OBS (110). Harmonic regression (111), another technique which is based on removing the spectral features of BCG, has shown similar or better results compared with AAS and OBS in experimental data. Morphological BCG removal (112, 113), which uses a Discrete Hermite Transform to model BCG shape, was shown to be superior to AAS and comparable to ICA, in the original paper in which it is described. Kalman filtering has been shown to be comparable to AAS, and may be preferred for real-time monitoring (114), but is unlikely to supersede AAS for post-processing, due to its computational complexity. Dictionary learning is able to model BCG well, but experimental data show that BCG removal was not significantly improved compared to the more common methods of AAS and OBS (116). Of all other methods for BCG removal, spectral and morphological methods appear to carry the most promise; however, all these approaches have not been validated independently outside of the group that first described them, making it difficult to recommend them given the current evidence.

##### 4.2.2.4. Comparisons of Post-processing Methods

The most commonly compared processes are AAS, OBS, and ICA, and to a lesser extent, clustering and CCA methods. Individual comparisons of OBS and AAS have shown conflicting results, with some favoring AAS (66, 107, 117), and others favoring OBS (28, 73), although the consensus is that it may depend on the research question or EEG correlate of interest, as to which method will provide the best reduction in BCG artifact, without losing possible EEG information of interest (118). A recent 7 T study showed that a k-means clustering algorithm may be a good trade-off between neuronal preservation and BCG attenuation compared with AAS and OBS (80), but no other studies have independently compared clustering algorithms. Considering that AAS is OBS with a PC number of 1, altering the number of PCs chosen as BCG artifact has been a source of investigation, with one study showing that OBS with a default PC number of 3 provides better BCG removal compared with AAS (76), while a later study showed no significant differences between AAS and OBS (PC = 3), unless the PC number chosen was optimized for each individual participant, case in which OBS outperformed AAS (73). Research has shown that for task-based EEG-fMRI studies involving high numbers of trials, use of AAS, OBS, or CCA is adequate for removing BCG (107), while for studies looking at fewer trials, or when EEG correlates are small and difficult to detect, BSS methods such as OBS (28) or CCA (107) may improve removal of BCG, and thus, detection of the underlying EEG correlate. Most studies show that ICA is best used after either AAS or OBS, because the difficulty of correctly choosing the number of components and ensuring stability of the algorithm means that the outcome might otherwise be more likely to involve erroneous results for little gain in BCG removal (76, 94, 119). Other methods, such as spatial, morphological, wavelet, dictionary, and spectral methods for reducing BCG have not been independently compared with OBS, AAS, or ICA outside of the original papers that introduced them, making it impossible to judge the quality and reliability of these methods.

#### 4.2.3. Direct Artifact Recording

There is evidence that the biggest contributor to BCG is pulse driven head motion, which obviously cannot be avoided during EEG-fMRI in human subjects (17, 75, 97). Options for measuring this physiological head motion artifact include methods to directly measure artifact at the head, such as altering the EEG cap, or using additional hardware connected to the cap. The benefits of these systems is that they are able to directly record any artifact occurring at the head, including small and large head motion, as well as monitoring environmental artifact such as vibration (120). However, higher costs and technical skill is required to ensure that any new and modified hardware is used safely and accurately.

Alterations to the EEG cap can include isolating some electrodes from the scalp using plastic (121–123), artificially joining electrodes using a gel bridge (124), or altering the position of some electrodes and bundling cables so that neuronal signals and motion can be adequately distinguished (125). The above options, however, result in sacrificing some electrodes for motion detection purposes, and may not be suitable for all EEG-fMRI setups, especially where electrode number is small or electrode number is important for the study outcome. Another method is to increase the number of EEG leads to provide an over-complete set of measurements that can be subsequently analyzed to distinguish between voltages arising from neuronal activity and those arising from motion of the sensors in a magnetic field (126). Further methods for alteration of the EEG cap include the creation of an entirely new electrode reference layer, isolated from scalp potentials, and located either underneath the EEG cap (127), on top of the EEG cap (128), or between EEG electrodes (129). For researchers not wanting to alter the EEG cap itself, additional sensors, such as extra electrodes (130), motion sensors (131), and carbon wire loops (55, 132–134), can be used to directly measure artifact from any sources including motion, without requiring alterations to the EEG cap itself. Signals obtained from direct artifact sensors can be successfully removed from the EEG with the aid of, for example, a multi-channel least squares fitting algorithm (55).

A study comparing the reference layer cap, wire loops, and AAS showed that while all methods adequately reduced BCG, using additional hardware (loops or reference cap) to filter data acquired during periods of deliberate motion, led to lower RMS values compared to AAS (120). This study suggested that the reference layer cap may be preferable to wire loops, but this was not shown definitively, as data obtained from the two methods were measured at different time points (120). While direct recording of artifact is preferable for all EEG-fMRI studies, implementation of this will depend on the research group's preferences and technical skill level.

#### 4.2.4. Recommendations for Removing Ballistocardiogram Artifact

**Recommendations for Reducing Ballistocardiogram Artifact in EEG-fMRI:**

- Additional hardware, such as carbon wire loops, additional sensors, or a reference layer cap, to directly measure BCG and motion:
  - Adaptive filtering to remove BCG based on direct recordings.
- Where additional hardware is not available, use of AAS, OBS or CCA or clustering methods with:
  - Recording of physiological signals (ECG, PPG, or respiration).
  - Precise recording and detection of QRS complexes using k Teager Energy Operator or Pan-Tompkins algorithm.
  - ICA post-processing of data to remove residual BCG if using AAS or OBS.

#### 4.2.5. Contemporary Use of BCG Removal Methods

**Figure 5:**
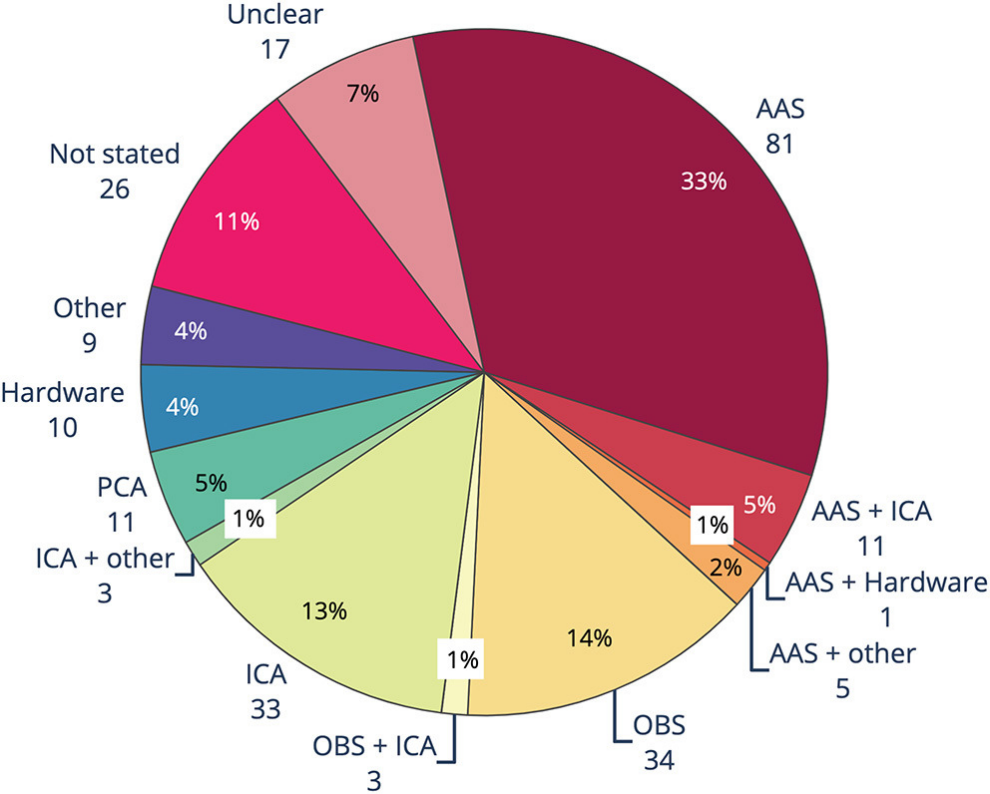
Ballistocardiogram (BCG) artifact removal method in literature using EEG-fMRI published between 2016 and 2019 (*n* = 244). AAS, average artifact subtraction; OBS, optimal basis set; ICA, independent components analysis; PCA, principle components analysis.

The main methods used to remove BCG artifact from EEG-fMRI studies between 2016 and 2019 consisted of AAS, OBS, ICA and PCA, as well as hardware methods such as direct recording of BCG by wire loops or modified EEG cap (Figure 5). Like GA removal, the most common method of reducing BCG was AAS, with around one-third of studies using AAS alone for removing BCG, compared to almost two-thirds using AAS for removing GA. Use of OBS was the second most common BCG removal technique, with 14% of studies, a slightly higher percentage than for GA removal (10%). ICA rivaled OBS as a popular form of BCG removal at 13%, with a further 2% of studies using either ICA and OBS, or ICA and another technique. Hardware (4%), PCA (5%), and other methods (4%) were less frequently adopted. BCG removal method was either unclear, or not stated, in 18% of studies.

The biggest difference seen between the methods for GA and BCG removal is the large uptake of BSS methods, particularly ICA and to a lesser extent PCA, for BCG removal (Figure 5). When ICA was used in conjunction with another method, total reported use of ICA was over 15%. Our guidelines recommend use of ICA only in conjunction with, and after, another method, due to the likelihood of either under- or over-removal of BCG (76, 94, 119).

BCG artifact is most commonly measured by separate ECG channel, with around two-thirds of studies included in the review reporting collection of ECG data (see Supplementary Material). In comparison, only 11% of studies reported using physiological monitoring such as a respiratory band or pulse. Nine percent of studies used external hardware, such as modified EEG caps, wire loops, and/or external sensors such as cameras, for measuring artifact occurring at the head (see Supplementary Material), yet of those, only 5% of studies reported using hardware for removing BCG (Figure 5). These results show that easy to implement techniques such as measuring ECG are more widely adopted than unique measures such as physiological monitoring and external hardware, despite the benefits of using external hardware. The technical expertise, money, time or skill in implementing additional artifact reduction hardware may be beyond the capacity of many researchers using EEG-fMRI.

Incorrectly removing artifact can lead to erroneous reporting of EEG results, incorrect mark-up of EEG events, and inaccurate EEG-fMRI outcomes (135). Eighteen percent of studies are unclear or do not state the BCG reduction method used. Considering the importance of reproducibility in science and the need to be able to replicate studies, it is important that artifact reduction methods are implemented and fully described in the Methods section.

### 4.3. Motion Artifact

Gross head motion is that which involves potentially large, sudden head movements as well as slow changes to head position. Reducing gross head motion can be achieved by using restraints such as foam padding or a vacuum cushion around the subject's head (86). Foam padding or vacuum cushions are readily available with most MR systems and should be used when available.

In subject populations where gross head motion is expected (such as children), the use of additional monitors for motion could be useful. During post-processing, these measurements of head motion can be used to filter out motion artifact or exclude periods of data if motion is considerable. Motion monitoring can be in the form of an Optical Motion Tracking system for whole head movement (45, 136) or indirect measurement of motion using an MR compatible camera (137). In addition, techniques used to directly measure BCG-related artifact (as outlined in section 4.2.3) can also be used to filter out gross head motion. A recent study (138) compared an Optical Motion Tracking system (MPT) (139) and two direct artifact recording methods: a reference layer cap (129) and isolation of electrodes from the scalp (123). The direct artifact recording measures both outperformed the MPT in reducing artifact during different tasks and different types of motion (138). Therefore, hardware to directly record artifact may be a good investment, as it is able to reduce both BCG and gross head motion artifact with greater accuracy than many data-driven methods (132, 136), and it may provide more accurate artifact estimation compared with motion detection hardware such as Optical Motion Tracking systems (138).

For studies where the subject has their eyes open, recording the EOG may be helpful for measuring eye movement (137). In the literature search conducted, no papers specifically dealt with removal techniques for EOG artifact from EEG-fMRI datasets. There are, however, many papers that describe removal techniques for EOG artifact from datasets recorded outside the MRI environment [for a recent review, see (138)]. These methods are likely also suitable for within-MRI recording, provided that GA and BCG artifact are adequately removed in previous steps. In the MRI scanner, the electrodes positioned near the orbits may exhibit additional localized motion artifact due to eye movement, as well as the concomitant EOG signal, potentially aiding in detection of such events.

#### 4.3.1. Recommendations for Reducing Motion Artifact

**Recommendations for Reducing Motion Artifact in EEG-fMRI:**

- Use of head restraints such as foam pads or vacuum cushion for all studies
- Additional hardware to measure motion should be considered:
  - In eyes open studies, use of EOG electrode to measure eye blink movement.
  - In all studies, direct artifact measurements, such as EEG cap alterations or additional sensors such as carbon fiber loops (see section 4.2.3), are recommended as they can deal with head motion, BCG, and residual GA. Indirect methods such as MR-compatible camera recordings or Optical Motion Tracking may also be useful for dealing with motion-related artifact.

#### 4.3.2. Contemporary Use of Motion Artifact Removal Methods

Almost 70% of contemporary studies mentioned removal of motion, or residual artifact that might be still present in EEG, even after GA and BCG removal (for all data, see Supplementary Material). Residual artifact, including motion, was dealt with by using some form of ICA, in around one-third of studies, while other data-driven post-processing methods for reducing motion were used in 15 % of studies. For reducing motion artifact exclusively, the most reported method, as seen in 23% of studies, was using head restraints such as foam pillows or a vacuum cushion. Also popular was the technique of removing artifact from studies by visual inspection of motion-related time points and excluding data during post-processing, which was reported in 16% of studies. The most common form of additional hardware reported was MR compatible cameras or video systems (4%), followed by motion loops and modified EEG caps (5% combined). A small percentage of studies used an external calibration of eye blinks to reduce artifact from eye movement in their studies. It is also worth noting that 5 studies, or 2%, reported that the subjects were sedated during the EEG-fMRI study, and therefore did not record motion. However, sedation does not remove BCG artifact or potential environmental sources of motion (see section 4.4), so we would recommend direct recording of head motion artifact even when subjects are sedated.

Compared with our recommendations, less than a quarter of studies have reported reducing motion through simple measures such as head restraints, for example, foam padding or vacuum cushions. Additionally, the uptake of hardware such as motion loops or modified EEG caps for recording motion are very limited, at only 5% of all studies. While a camera or video monitoring system (4% of all studies) can be good for indirectly measuring motion and therefore excluding periods of high motion, direct motion monitoring systems allow the user to directly record, and remove artifact directly occurring at the head. A greater uptake of direct recording systems in the future could allow an improvement in EEG-fMRI studies through reduction of motion artifact.

### 4.4. Environmental Artifact

While the noise from gradient, cardio-ballistic, and subject motion are the main sources of artifact in EEG-fMRI, noise from the MRI environment such as power line noise, lights, and ventilation are also a problem that few studies have addressed (18). Of all the possible environmental sources of noise, the ventilation system and helium cooling pump have been shown produce the most noise (33), which is thought to be mainly due to small vibrations that induce motion, and therefore voltage, in EEG electrodes. Environmental artifact can be avoided by turning off electrical equipment which is unnecessary during scanning, if possible and safe to do so (18). Artifacts can also be reduced by altering the setup of some equipment, specifically by reducing the vibration artifact on EEG cabling. EEG leads and amplifiers should be isolated from the scanner bore in order to reduce direct transfer of vibration, and methods such as using sand or rice bags (86, 140), or a cantilever beam (141), will improve stability and restrict the motion of the EEG cables connecting the EEG cap and amplifiers.

For setups where the helium pump and ventilation cannot be turned off for the duration of the study (e.g., due to safety concerns or the length of the scan), and environmental artifact is shown to negatively affect the quality of the EEG, the use of direct artifact recording, or post-processing methods for removing environmental artifact should be considered. Template measures such as AAS have been shown to reduce ventilation and helium cooling pump noise compared with no intervention (142), while another study has shown that a BSS algorithm, recursive, Segmented PCA (rsPCA), is able to reduce MRI helium pump noise (14). Alternatively, direct artifact recording measures, such as carbon wire loops (section 4.2.3), have also been shown to adequately remove helium pump artifacts, with filtering using carbon wire loops showing less residual helium pump artifact in comparison to AAS, OBS, or OBS-ICA artifact reduction pipelines (132). While altering the setup of the EEG-fMRI study to reduce vibrational artifact is relatively easy to implement, the choice to include additional hardware or post-processing steps for reducing MR helium pump and ventilation noise will depend on the residual artifact seen in the individual scanner setup, and whether it is likely to adversely affect the outcome of the study.

#### 4.4.1. Recommendations for Reducing Environmental Artifact

**Recommendations for Reducing Environmental Artifact in EEG-fMRI:**

- EEG leads and amplifiers should be secured along the center of the bore using one of:
  - Sand or rice bags, or
  - Cantilever beam (141)
- If safe to do so, MR helium cooling pump and scanner ventilation could be turned off for the duration of the scan.
- If environmental artifact is expected to interfere with the EEG of interest, using carbon wire loops (55, 132) will directly record this artifact, enabling its removal via adaptive filtering.
  - If carbon wire loops are not available, using AAS or PCA methods during post-processing may help reduce residual environmental artifact.

#### 4.4.2. Contemporary Use of Environmental Artifact Reduction Methods

In contemporary literature of EEG-fMRI studies, consideration of environmental artifact was reported in only 22% of studies (see Supplementary Material), with the few studies reporting mitigation of environmental artifact using methods to reduce EEG cable vibrations (7 %) and either: optimizing amplifier placement, or turning off lights during the experiment (11%). Despite literature suggesting that the helium cooling pump and scanner ventilation system may affect EEG-fMRI recordings (18), only around 5% of studies report taking steps to turn off, measure, or reduce, the effects of these artifacts, respectively. Only one study in the cohort reported use of a post-processing method (13) for removing helium pump artifact. Although environmental artifact may be reduced during other post-processing methods for removal of GA and BCG, and environmental artifact may not interfere with the EEG correlate of interest in the study, there appears to be a lack of awareness of this potential artifact within the wider research community.

### 4.5. Artifact Removal Toolboxes: Contemporary Usage

**Figure 6:**
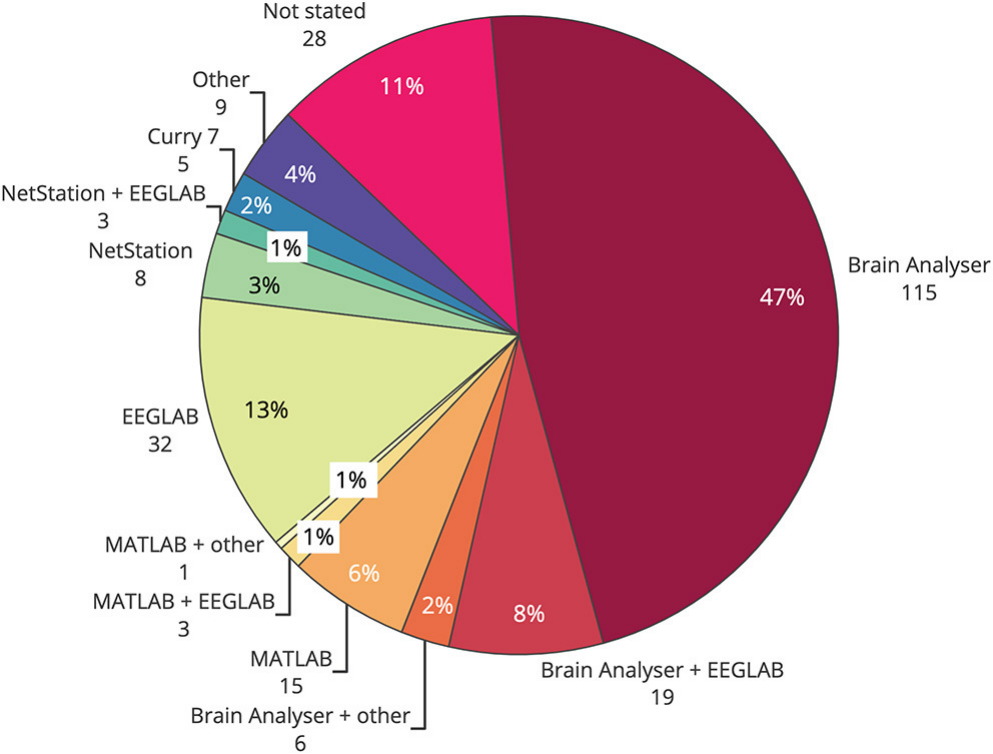
Use of software toolboxes for filtering artifact from EEG collected during fMRI, papers published 2016–2019 (*n* = 244).

Commercial EEG-fMRI systems may influence the choice of artifact reduction method used in any particular study. An overview of common toolboxes, and where to find them, is presented in Table 1. Brain Vision Analyser, from Brain Products, was the most widely used single toolbox for removal of artifact in EEG-informed fMRI (Figure 6), with over half of studies published using the software for at least part of the filtering. EEGLAB, a toolbox requiring MATLAB, was the second most used software, with over 20% of all studies reporting its use in some part of their processing. Given that Brain Vision Analyzer as standard employs AAS for gradient and BCG correction, and that OBS is available as a plug in for EEGLAB (FMRIB plug in), the results of toolbox use show a similarity between the use of methods and their software. Given that most researchers are time poor and may not have the technical expertise to test different artifact reduction methods, the most commonly used methods are the ones that are readily available and easy to access. Therefore, it is crucial that developers of new EEG-fMRI artifact removal algorithms consider the functionality and ease of use for the end users of any new software or algorithms.

**Table 1:** Examples of commonly cited toolboxes for removing artifact from EEG recorded during fMRI.

**Toolbox**	**Manufacturer**	**Cost**	**Web link**	**Notes**
Brain Vision Analyzer	Brain Products	Paid software, requires license	https://www.brainproducts.com/analyzer2_release.php	Standalone software for processing EEG. Typically purchased together with BrainProducts hardware.
Netstation	Magstim EGI	Paid software, requires license	https://www.egi.com/research-division/net-station-eeg-software	Standalone software for processing EEG. Typically purchased together with Magstim EGI hardware.
Curry	Compumedics Neuroscan	Paid Software, requires license	https://www.compumedics.com.au/en/products/curry/	Standalone software for processing EEG. Typically purchased together with Compumedics hardware.
EEGLAB	Swartz Center for Computational Neuroscience, University of California	Free and open source, requires Matlab (see below)	https://sccn.ucsd.edu/eeglab	Free toolbox for MATLAB, contains many useful EEG filtering tools, as well as additional plug ins specific to filtering fMRI artifact from EEG. Toolboxes include: • FMRIB plug in (http://fsl.fmrib.ox.ac.uk/eeglab/fmribplugin/) • BERGEN (may no longer be supported, still listed on the EEGLAB plug-in site).
MATLAB	Mathworks	Paid software, requires license	https://au.mathworks.com/products/matlab.html	General analysis software environment. User can write their own code for filtering the EEG, or combine custom code with the output from EEGLAB toolbox (see above).

### 4.6. Overall Recommendations for Artifact Reduction in EEG-fMRI

**Figure 7:**
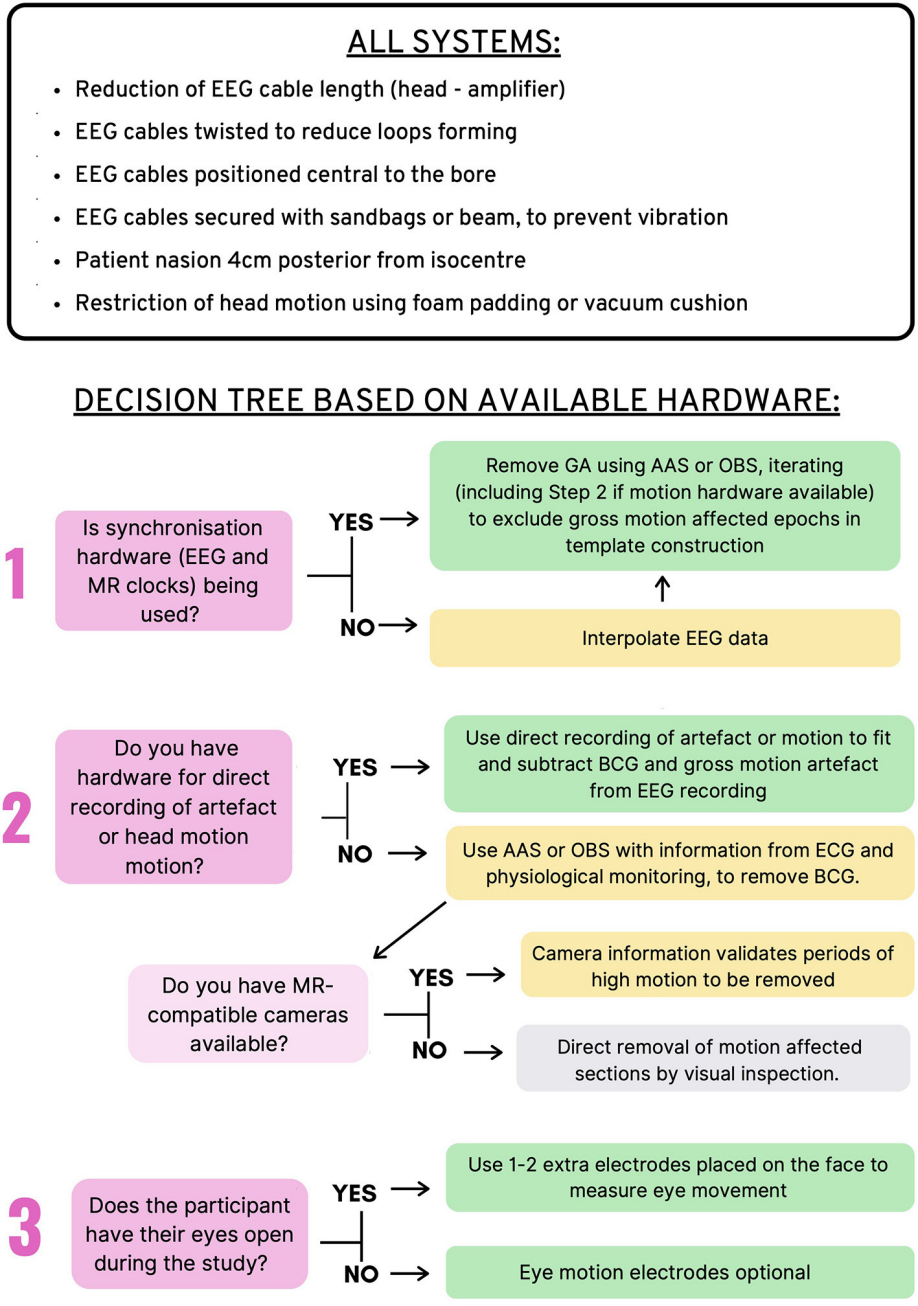
Recommendations for Removal of artifact in EEG-fMRI studies. **Top:** Recommendations for all EEG-fMRI setups; **Bottom:** Recommendations based on hardware available and study design.

Figure 7 provides an overview of the recommendations for EEG-fMRI artifact removal based on the current literature review. These recommendations include steps that all EEG-fMRI users should take to avoid artifacts in the recording, as well as 3–4 questions that users can ask to make the best possible decisions for removing artifact during post-processing, depending on the study design and hardware available. These recommendations represent the combination of all recommendations from previous sections of this review (gradient, BCG, motion, and environmental artifact).

A limitation of the current study is that there is no distinction between the so-called “online” and “offline” artifact reduction techniques. Online artifact reduction refers to the filtering of artifact from EEG recording during fMRI in as close to real time as possible, whereas offline artifact removal undertaken at the conclusion of the data collection. For this review, it is assumed that the EEG-fMRI study is designed in such a way that the researcher has ample time to filter and interpret EEG (offline artifact removal), gaining results from the study well after the subject has left the room. However, for certain study designs, such as neurofeedback studies (143), clean EEG is required with as little lag time as possible, so that data are available during the study, and therefore, online reduction is required. Several studies have adapted common artifact reduction methods for real-time use, including AAS (144, 145), OBS (92), and FT (68). Online artifact reduction prioritizes computational efficiency and speed over accuracy of the artifact reduction on EEG, and therefore, some recommendations given in this review will not be relevant for studies of this design.

Another aspect of the current review not yet discussed is that the EEG measure of interest varies between different studies. In our survey of contemporary EEG-fMRI literature, the most popular EEG measurements were epilepsy-related EEG events, event-related potentials, and EEG power bands (see section 6.5 in Supplementary Material for more detailed information). This creates a question as to whether there is a need for high level artifact removal in all studies, or whether basic removal of artifacts may be enough to see the EEG measure of interest. There is, however, always a risk that using sub-optimal artifact removal will result in false positive EEG features being reported. Evidence from literature shows, for example, that marking motion in EEG as though it is an effect of interest may lead to plausible looking networks in the subsequent fMRI analysis (135). We recommend that EEG-fMRI users should employ the best possible EEG setup and artifact removal possible to reduce the chances of false positive EEG features being reported. While not all scientists will have access to the hardware components mentioned in Figure 7, we have endeavored to include alternative methods, so that all users of EEG-fMRI can have the best chance of producing accurate and reproducible results.

## 5. Conclusions

There are many published artifact removal methods for EEG-fMRI, however, uptake of newer methods in contemporary studies is limited. In addition, many novel methods have been proposed that have not been adequately compared with the currently available methods on independent datasets. Our review provides a comprehensive overview of all artifact reduction methods available since 1998 (see Supplementary Material), and there is an opportunity for future studies to compare these different methods in one comprehensive data set to independently validate the best pipeline for artifact reduction in EEG-fMRI.

Best practice EEG-fMRI artifact reduction relies largely on using additional hardware to synchronize clocks, and to directly record artifact at the head, including motion, vibration, and eye blinks. Without monitoring from hardware during EEG-fMRI studies, the researcher is making assumptions as to which aspects of the recording represent artifact, and which represent plausible neuronal activity. However, our review of the contemporary literature showed that reported use of synchronization hardware cited in less than 50% of studies, and reported use of hardware to directly measure artifact was even less frequently cited (less than 10% of studies, see Supplementary Material). Actual use may be higher in practice, but cannot be included in these statistics due to lack of reporting in the literature. Software used for filtering EEG showed a preference for BrainVision Analyser and EEGLAB, and it is likely that the ease of use of these packages played a role in their wide use. Therefore, it can be hypothesized that the lack of accessibility of direct artifact recording measures is hampering their use in contemporary studies, despite literature showing that their use may result in more accurate EEG-fMRI studies (120). Compared with synchronization hardware, which is included in some commercial EEG-fMRI setups, direct artifact recording has not been available commercially, leaving it up to researchers to bear the cost should they wish to directly record artifact using hardware.

Overall, there appears to be a basic understanding in contemporary EEG-fMRI studies about the importance of reducing artifact, especially for gradient and BCG artifacts. However, there was almost 15% of contemporary studies that did not adequately report artifact reduction methods for GA and BCG, and very few studies reported methods to avoid or reduce motion and environmental artifacts at all. A review such as this, and indeed reproducible science, is limited by the extent to which researchers accurately and completely report their methods. We would encourage authors publishing EEG-fMRI results to fully report all measures taken to reduce artifact to help make their research as reproducible as possible. We recommend authors consider the recommendations from the Organization for Human Brain Mapping's Committee on Best Practices in Data Analysis and Sharing (COBIDAS) for reproducible EEG and MEG research (146). If a journal word-count limit makes this difficult in a main manuscript, then authors should consider including detailed methods in Supplementary Information.

This review provides recommendations for artifact reduction in EEG-fMRI in an easy to understand way for all users of EEG-fMRI (see Figure 7). We suggest that wider use of hardware methods such as clock synchronization and direct artifact recording is the easiest way to improve data accuracy in EEG-fMRI studies, but seeing adoption of hardware methods requires input from all EEG-fMRI stakeholders—from methods developers, vendors, and end users.

## Data Availability Statement

The original contributions generated for the study are included in the article/Supplementary Material, further inquiries can be directed to the corresponding author/s.

## Author Contributions

MB conducted the literature search. MB and DA contributed to the manuscript preparation. MB, DA, and GJ revised the manuscript. All authors read and approved the final manuscript.

## Conflict of Interest

The Florey Institute of Neuroscience and Mental Health acknowledges a partnership with Brain Products GmbH toward development of commercially available carbon wire loops (CWL) for direct artifact detection and correction. This includes work that was in part supported by an Australian Government Global Connections Fund Bridging Grant (Application number 279313678, awarded to DA). The authors declare that the research was conducted in the absence of any other commercial or financial relationships that could be construed as a potential conflict of interest.
