# Supplementary material for: Artifact Reduction in Simultaneous EEG-fMRI: A Systematic Review of Methods and Contemporary Usage
Source: Front Neurol. 2021 Mar 11;12:622719. doi: 10.3389/fneur.2021.622719 (PMC7991907; doi:10.3389/fneur.2021.622719)
Supplement: Supplementary file 1 [file Data_Sheet_1.pdf]

Supplementary Material

1 GRADIENT ARTEFACT REMOVAL METHODS

Table S1: Methods for Reducing Gradient Artefact (GA) in Simultaneous EEG-fMRI Recordings, Literature 1998-2018.

Method	Description	Literature
**Avoiding GA**		
Stepping stone sampling (SSS)	Novel fMRI sequence, where EEG is sampled between slices of fMRI, thus avoiding GA. Requires accurate synchronisation of MR and EEG clocks.	Anami et al. (2003)
MR-Link Device	Wireless amplifier that reduces the effect of electromagnetic interference during recording. Currently only tested in animal MRI.	Mandal et al. (2019)
**Reducing GA**		
Short cable length	Reducing the overall length of the cables reduces induced voltage from GA reaching EEG amplifiers, due to reduction in size of loops of EEG cables in the magnetic field.	Chowdhury et al. (2015)<br>Assecondi et al. (2016) *
Twisted EEG cables (cap)	Twisting cables from opposite sides of the EEG cap together reduces GA, due to reduction in cable loop size and noise caused by gradient and radiofrequency (RF) pulses during fMRI.	Allen et al. (1998)<br>Goldman et al. (2000)
Twisted leads (cap to amplifier)	Twisted leads outperform flat ribbon leads when connecting EEG cap to the amplifier inside the MR environment, due to reduction in loop size in the magnetic field.	Chowdhury et al. (2015)<br>Jorge et al. (2015a)
Cable position on EEG cap	Positioning the cable bundle at Cz electrode when leaving the EEG cap, could reduce GA effects over the temple region; although this has not been conclusively validated in human subjects.	Mullinger et al. (2014)
Novel EEG cap	High density (256 channels) EEG cap (Inknet) showed reduced GA on 3T fMRI images, compared with similar high-density copper wire EEG cap.	Poulsen et al. (2017) **
Re-wiring EEG cables	Altering the configuration of EEG leads to reduce scanner specific GA contamination.	Chowdhury et al. (2019)
		*4T study , **7T study

Table S1: Methods for Reducing Gradient Artefact (GA) in Simultaneous EEG-fMRI Recordings, Literature 1998-2018.

Method	Description	Literature
**Clock Synchronisation**		
Synchronisation of EEG and MRI clocks	Synchronisation of EEG and MRI clocks using specialised hardware, helps with reducing residual GA during post processing of EEG recordings, when template subtraction methods are used.	Anami et al. (2003)<br>Mandelkow et al. (2006)<br>Mullinger et al. (2008a)<br>Gebhardt et al. (2008)
EPI fMRI sequence	Echo-planar imaging (EPI) fMRI sequence produces less GA on EEG than spiral fMRI sequence. GA from both sequences can be mitigated by appropriate AAS and clock synchronisation.	Solana et al. (2014)
Post processing synchronisation	Use of:<br>(a) interpolation ,<br>(b) time continuous cubic spline model,<br>(c) interpolation and auto-correlation , and<br>(d) least across squared variance<br>methods for correcting for datasets where EEG and MR clocks are not synchronised.	(a) Goncalves et al. (2007a,b)<br>(b) Koskinen and Vartiainen (2009)<br>(c) Mandelkow et al. (2010)<br>(d) Tan et al. (2017)
**Post-Processing: Template Methods**		
Average Artefact Subtraction (AAS)	Segmentation of EEG data into epochs based on MR image acquisition trigger. An average template is created for each of the epochs and interpolation is used to remove this artefact template from the EEG.	Allen et al. (2000)
"Real time" (online) AAS	Adapted AAS algorithm to calculate imaging artefact in close to real time, to allow for reading EEG during fMRI scan and immediate feedback.	Garreffa et al. (2003);<br>Gualniera et al. (2004)
Additional information for AAS	(a) Inclusion of head displacement parameters from analysis of fMRI images to improve the estimation of GA template.<br>(b) External sensors used to estimate motion and use in calculation of GA template.	(a) Moosmann et al. (2009)<br>Sun et al. (2009)<br>(b) Zhang et al. (2019)

Table S1: Methods for Reducing Gradient Artefact (GA) in Simultaneous EEG-fMRI Recordings, Literature 1998-2018.

**Method**	**Description**	**Literature**
AAS extensions	Additional steps added to AAS for GA removal:<br>(a,b) Addition of adaptive filter to minimise residual GA after AAS<br>(c) Weighting artefact templates based on proximity<br>(d) Amplitude of each template subtraction adjusted by linear regression<br>(f) Cross correlation used to phase shift each template to optimally fit each epoch<br>(g) Pre-process EEG with non-linear filter and signal slope adaptation (SSD) prior to AAS	(a) Allen et al. (2000) (adaptive noise cancellation)<br>(b) Wan et al. (2006a)<br>(c) Freyer et al. (2009)<br>(e) Sartori et al. (2010)<br>(f) Huang et al. (2012)<br>(g) Ferreira et al. (2014)
Basis Set of Templates	A set of artefact templates based on cubic spline interpolation. Each epoch's artefact is defined as a combination of templates from the set.	LeVan et al. (2016)
Hierarchical clustering	Many templates are created based on data from the whole recording. Epochs are grouped into similar clusters, and templates are applied to clusters rather than using a sliding window.	de Munck et al. (2013)
**<u>Post-Processing: Blind Source Separation Methods</u>**		
Independent Component Analysis (ICA)	Separates the signal into Independent Components (IC), based on ICs being as non-Gaussian as possible.	Ryali et al. (2009)
Independent Vector Analysis (IVA)	ICA using multiple datasets, with each EEG channel considered a separate dataset.	Acharjee et al. (2014, 2015)
Principle Component Analysis (PCA)	Reduces dimensionality by maximising variation in the data set. Results in a set of Principal Components (PC), with the first PC explaining most of the variability in the data set. PCs estimated to be related to GA are removed.	Negishi et al. (2004)
Optimal Basis Set (OBS) / FASTR.	After template subtraction, PCA is used to determine an OBS. The PCs from the OBS that relate to artefact are removed.	Niazy et al. (2005)
Single Value Decomposition (SVD)	One form of PCA. SVD decomposes a mixed matrix into orthonormal matrices, which can be ordered by variance.	Liu et al. (2012)

Table S1: Methods for Reducing Gradient Artefact (GA) in Simultaneous EEG-fMRI Recordings, Literature 1998-2018.

Method	Description	Literature
Canonical Correlation Analysis (CCA)	Maximising temporal correlation across the data set, obtaining components which are uncorrelated and represent artefact.	Li et al. (2017)
Blind Source Extraction (BSE)	BSS technique, with aim to separate one source of interest, therefore limiting number of sources to be found.	Jing and Sanei (2006)
**Post-Processing: Other Methods**		
Frequency filtering	(a) Removing artefact from EEG based on its frequency components<br>(b) Real time filtering using the Fast Fourier Transform<br>(c) Taylor-Fourier Transform: An extension of the Fourier Transform that takes into account more frequencies, is applied to GA at each individual TR	(a) Hoffmann et al. (2000)<br>(b) Shaw (2017)<br>(c) Frigo and Narduzzi (2014)
Dictionary Learning	Mixed over complete Dictionary (MOD), containing wavelets and discrete cosine functions, sparsely represents the EEG and GA. Matching pursuit algorithm (MP) separates EEG and GA signals from the dictionary.	Xu et al. (2005)

2 BCG ARTEFACT REMOVAL METHODS

Table S2: Methods for Reducing Ballistocardiogram Artefact in Simultaneous EEG-fMRI Recordings, Literature 1998-2018.

Method	Description	Literature
**Avoiding BCG**		
ECG triggered stimulus delivery	Use of ECG channel to estimate BCG artefact peaks that will be seen in EEG. Deliver task (stimulus) during times of low BCG artefact to reduce its impact on EEG signal.	Ertl et al. (2010)
**Monitoring Physiological Signals**		
ECG electrodes	An additional electrode placed on the back or chest measures echocardiogram (ECG) signals, which are collected for the purpose of estimating BCG.	Allen et al. (1998)<br>Goldman et al. (2000)

Table S2: Methods for Reducing Ballistocardiogram Artefact in Simultaneous EEG-fMRI Recordings, Literature 1998-2018.

**Method**	**Description**	**Literature**
Scanner equipment	Scanner inbuilt photoplesythmography (PPG) and respiration band is used to measure the heart rate and breathing rate of the subject, with data used for later estimation of BCG artefact.	Mullinger et al. (2008a)
**Direct Artefact Recording: EEG Cap Alterations**		
Multi lead EEG	A custom multi-lead EEG cap oversamples the EEG, artefact is removed during post-processing based on spatial voltage differences.	Dyrholm et al. (2009)
Dual array EEG	Modified commercial brain cap, by moving some electrode positions, to produce orthogonal loops that detect head motion. ICA used post-recording to remove motion artefact.	Klovatch-Podlipsky et al. (2016)
Conductive gel bridge sensor	Using the dual array EEG layout (above), but applying the conductive gel between electrodes to form an artificial bridge. Affected electrodes used as a motion sensor.	Cohen et al. (2019)
Isolation of electrodes	Use of plastic layer (ie. plastic tape, shower cap) for isolating a subset of electrodes from a high density (256 channel) cap. Isolated electrodes are used to provide an estimate of motion artefact.	Xia et al. (2013a,b, 2014a,b)<br>Jorge et al. (2015b) **
Artefact reference layer	Use of an additional "reference" layer of electrodes which are isolated from the scalp, either placed:<br>(a) on top of<br>(b) or underneath the EEG cap<br>(c) or between EEG electrodes<br>The reference layer records general artefact occurring at EEG electrodes, including motion artefact, which can be removed directly from EEG during post-processing.	(a) Chowdhury et al. (2014),<br>Chowdhury et al. (2019)<br>(b) Luo et al. (2014)<br>(c)Steyrl et al. (2015, 2017, 2018)
**Direct Artefact Recording: Additional Sensors**		
Piezoelectric sensor	Piezoelectric sensor attached to subject's temple measures motion, filtered from EEG with adaptive noise cancellation.	Bonmassar et al. (2002)

Table S2: Methods for Reducing Ballistocardiogram Artefact in Simultaneous EEG-fMRI Recordings, Literature 1998-2018.

Method	Description	Literature
Facial electrodes	Use of high-density EEG and facial and temporal electrodes to determine BCG artefact due to small head motion. Difference between right and left side of non-neuronal electrodes showed better result for removing BCG than recording ECG.	Iannotti et al. (2015)
Wire loops	Large (∼10cm) copper or carbon wire loops attached to the outside of the EEG cap or sewn to the cap. Regression algorithm used during post-processing to reduce motion and ballistocardiogram artefacts from EEG.	Masterton et al. (2007); Abbott et al. (2014)<br>van der Meer et al. (2016a,b)
**<u>Post-Processing: Template Methods</u>**		
Average Artefact Subtraction (AAS)	Similar to AAS for GA (described above), with the exception that the segmentation of EEG data into epochs is based on R peaks of the QRS complex seen on ECG recordings, rather than MR triggers.	Allen et al. (1998)
AAS extensions	Additional steps added to AAS for BCG removal:<br>(a) Template scaled by exponentially updated weights, giving more weight to temporally close epoch templates<br>(b) After AAS, residual noise reduction by wavelet decomposition and RLS adaptive filtering<br>(c) Dynamic time warping. Correcting for slight temporal shifts in BCG by linearly warping the data to the template	(a) Goldman et al. (2000)<br>(b) Kim et al. (2004)<br>(c) Kustra et al. (2008)
BCG template optimisation for AAS	Algorithms to enhance the detection of cardiac R peaks, or BCG artefact template, used with AAS.	Ellingson et al. (2002, 2004)<br>Oh et al. (2007, 2014)<br>Wen et al. (2016), Wong et al. (2018)
Basis Set of Templates	A Fourier set of BCG templates, derived from the whole data set. BCG at each epoch is defined as a combination of templates from the set.	Vincent et al. (2007)
Hierarchical clustering	See previous section: Gradient Artefact.	de Munck et al. (2013)
**<u>Post-Processing: Blind Source Separation Methods</u>**		

Table S2: Methods for Reducing Ballistocardiogram Artefact in Simultaneous EEG-fMRI Recordings, Literature 1998-2018.

Method	Description	Literature
Independent Component Analysis (ICA)	See previous section: Gradient Artefact.	Bénar et al. (2003)
IC classification	Methods for classifying IC as neuronal activity or BCG artefact:<br>(a) Clustering ICs based on multiple ICA runs<br>(b) ICs sorted by minimum spectral difference to measured ECG<br>(c) Discrete Hermite Transform<br>Mutual Information algorithm:<br>- (d) with single value decomposition (SVD)<br>- (e) with a threshold criteria<br>(f) Correlation to ECG (maximum squared coherence)<br>(g) K means clustering (PROJIC) with additional OBS or AAS on BCG ICs<br>(h) Clustering ICs and removal based on spatial maps	(a) Briselli et al. (2006)<br>(b) Koskinen and Vartiainen (2008)<br>(c) Ferdowsi et al. (2012a)<br>(d) Liu et al. (2012)<br>(f) Silva de Souza et al. (2013)<br>(e) Abbasi et al. (2015)<br>(g) Abreu et al. (2016) **<br>(h) Piorecky et al. (2019)
Constrained ICA (cICA)	Converges IC to some prior estimation of noise, rather than being statistically independent.<br><br>Extension:<br>(a) Clustering of ICs from cICA for BCG classification	Rasheed et al. (2006, 2009)<br>Leclercq et al. (2009)<br>(a) Wang et al. (2018)
Principal Component Analysis (PCA)	See previous section: Gradient Artefact.<br><br>Extensions:<br>(a) Maximum Noise Fraction. Noise whitening with PCA<br>(b) Empirical Mode Decomposition (EMD) with PCA	Bénar et al. (2003)<br>(a) Sun et al. (2009)<br>(b) Javed et al. (2014a,b, 2015, 2017)
Optimal Basis Set (OBS) / FASTR.	See previous section: Gradient Artefact.<br><br>Extensions:<br>(a) Real time filtering<br>(b) Using prior information	Niazy et al. (2005)<br>(a) Wu et al. (2016)<br>(b) Marino et al. (2018)
Canonical Correlation Analysis (CCA)	See previous section: Gradient Artefact	Assecondi et al. (2008, 2009)

Table S2: Methods for Reducing Ballistocardiogram Artefact in Simultaneous EEG-fMRI Recordings, Literature 1998-2018.

Method	Description	Literature
Blind Source Extraction (BSE)	See previous section: Gradient Artefact	Kosma et al. (2009)<br>Ferdowsi et al. (2012b, 2013)
		** study included 7T data
**Post-Processing: Other Methods**		
Spatial	Multiple Source Correction. Topography of BCG recorded outside of the scanner used to cancel BCG from inside the scanner.	Siniatchkin et al. (2007)
Spectral	Statistical methods are used to estimate the main spectral properties of BCG.	Ghaderi et al. (2010)<br>Krishnaswamy et al. (2013, 2016)
Wavelet	Decomposition by a pre-defined wavelet function, which may help separate artefact or activity.	Wan et al. (2006b)
Dictionary	Mixed over complete dictionary sparsely models BCG signal.	Abolghasemi and Ferdowsi (2015)
Kalman	Iterative filter, which predicts and updates weights throughout.	In et al. (2006)<br>Sameni et al. (2008)
Morphological	Use of Discrete Hermite Transform (DHT) to separate artefact from activity based on the shape content of each signal.	Mahadevan et al. (2008a,b)

3 OTHER ARTEFACT REMOVAL METHODS

Table S3: Methods for Reducing Ballistocardiogram Artefact in Simultaneous EEG-fMRI Recordings, Literature 1998-2018.

Method	Description	Literature
**Reducing Head Motion**		
Head restraints	Vacuum cushion (plastic bag filled with polystyrene spheres, additional air removed by suction) successfully used to reduce head motion and improve patient comfort.	Bénar et al. (2003)

Table S3: Methods for Reducing Ballistocardiogram Artefact in Simultaneous EEG-fMRI Recordings, Literature 1998-2018.

**Method**	**Description**	**Literature**
EOG electrode	Electrooculogram (EOG), an electrode placed on the temple, is used to record eye blink movements, in an eyes-open visual EEG-fMRI study. Recordings from EOG electrodes can be used to remove eye blink artefact during post processing.	Bonmassar et al. (1999)
MR compatible camera	MR compatible camera mounted to the head coil during EEG-fMRI scan allows for post-recording exclusion of EEG data due to motion.	Ruggieri et al. (2015)
Optical motion tracking	Optical motion tracking system. Using Moiré Phase Tracking (MPT) marker attached to base of forehead and an MRI compatible camera, head motion can be measured with 6 degrees of freedom. Linear regression of the motion parameters reduces BCG and motion artefact in the EEG recording.	LeVan et al. (2013, 2016)
**Reducing Environmental Artefact**		
Restricting EEG lead and amplifier motion	Minimise EEG lead/amplifier motion:<br>- sand/rice bags<br>- cantilever beam	Kruggel et al. (2000)<br>Bénar et al. (2003)<br>Mullinger et al. (2008b)
AAS	See Part 1 - Gradient Artefact.<br>- Use of AAS for ventilation and helium cooling pump noise.	Rothluebbers et al. (2013)
PCA	Recursive, Segmented PCA (rsPCA) for MRI helium pump noise.	Kim et al. (2015)
**Other Post-processing Methods**		
ICA	See part 1- Gradient Artefact.<br>- Real time ICA, for non GA related artefact (BCG and mixed)	Mayeli et al. (2015, 2016)
Functional Source Separation	ICA constrained by an estimate of the noise, at the cost function level of the algorithm.	Porcaro et al. (2010)
PCA	See Part 1 - Gradient Artefact.<br>- (stepping stone acquisition, residual GA after AAS)	Freyer et al. (2009)

Table S3: Methods for Reducing Ballistocardiogram Artefact in Simultaneous EEG-fMRI Recordings, Literature 1998-2018.

**Method**	**Description**	**Literature**
Beamformer spatial filter	Estimation of the topographical artefact across EEG channels.	Brookes et al. (2008, 2009)**
		** 7T study

4 EEG-FMRI SETUP IN CONTEMPORARY STUDIES

Table S4: EEG-fMRI Setup: Contemporary Studies (2016-2018)

**Setup of EEG-fMRI**	**No. of papers**	**Percentage (%)**
**EEG channel number**		
- under 32 channels	75	31
- 32	31	13
- 33-63	57	23
- 64	49	20
- 65-100	5	2
- 128+	17	7
- Unclear	2	1
- Not stated	5	2
- Multiple	3	1
**Other channels**		
ECG	162	66
EOG	54	22
EMG	7	3
**Physiological Monitoring**		
Total, any monitoring (may use multiple techniques)	28	11
- Respiratory band	24	10
- Pulse oximetry	18	7
- Scanner Vectorcardiogram	6	2
- Chest accelerometer	2	1
**EEG / MRI clock synchronisation**		
Total using synchronisation	119	49

Table S4: EEG-fMRI Setup: Contemporary Studies (2016-2018)

**Setup of EEG-fMRI**	**No. of papers**	**Percentage (%)**
- Brain Products SyncBox	54	22
- MR trigger timed (TTL)	9	4
- Other device	2	1
- Device not stated	54	22
**Environmental Artefact**		
Total reporting on environmental variables	56	23
- Helium pump off	12	5
- Ventilation low / off	7	3
- EEG cables secure (e.g. sandbags)	16	7
- Other (e.g. lights off, amplifier configuration)	29	12
**Motion Artefact**		
Total reporting on motion artefact	165	68
- ICA for removing residual artefacts, including motion artefact	77	32
- Head cushioning / immobilisation	56	23
- Visual inspection of motion on EEG	41	17
- Data driven post-processing methods	15	6
- External calibration of eye blinks	5	2
- Camera / video system	10	4
- Other Hardware (modified EEG cap, motion loops)	11	5
- Sedation	5	2

5 ROLE OF EEG IN CONTEMPORARY EEG-FMRI STUDIES

For EEG-fMRI studies published between 2016 and 2018, the most common use of EEG data recorded with fMRI was for detection of epileptic events (epilepsy spikes), as shown in figure S1. This result is perhaps not surprising, given that the earliest iterations of EEG-fMRI during the 1990s used spike-triggered scanning to help localise epileptic regions in patients Warach et al. (1996). However, just under 50% of contemporary EEG-fMRI studies used EEG to measure either event related potentials (an EEG correlate of a task based activity), or power bands (EEG magnitude in a particular frequency range). Another 13% of studies used the EEG primarily for either sleep staging or determining spatial topology of an event in EEG. Finally, studies included in the ‘other’ section (n=15) included measures such as EEG: phase Onojima et al. (2017), microstates , speech envelopes Anwar et al. (2016), and measures of working memory Baenninger et al. (2016), or connectivity Anwar et al. (2016); Wirsich et al. (2017). The results of this review show that whilst simultaneous EEG-fMRI is still useful in studies involving epilepsy patients, its use has grown to include research into the fMRI activity during many different types of EEG events.

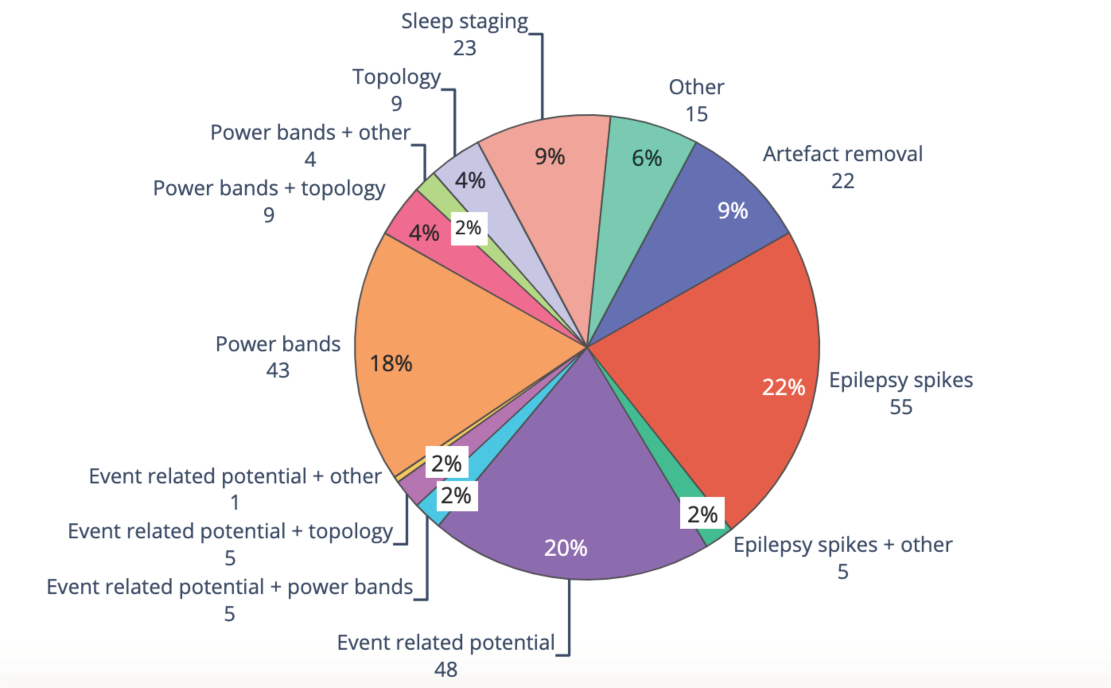

Figure S1. EEG measurements used during EEG-fMRI; papers published between January 2016 – December 2019 (n=244). IED = interictal epileptic discharge; ERP = event related potential.

REFERENCES

Anami K, Mori T, Tanaka F, Kawagoe Y, Okamoto J, Yarita M, et al. Stepping stone sampling for retrieving artifact-free electroencephalogram during functional magnetic resonance imaging. *NeuroImage* **19** (2003) 281–295.

Mandal R, Babaria N, Cao J, Liu Z. Adaptive and Wireless Recordings of Electrophysiological Signals During Concurrent Magnetic Resonance Imaging. *Ieee Transactions on Biomedical Engineering* **66** (2019) 1649–1657. doi:10.1109/TBME.2018.2877640. WOS:000468432000017.

Chowdhury MEH, Mullinger KJ, Bowtell R. Simultaneous EEG-fMRI: evaluating the effect of the cabling configuration on the gradient artefact. *Physics in Medicine and Biology* **60** (2015) N241–250. doi:10.1088/0031-9155/60/12/N241.

Assecondi S, Lavallee C, Ferrari P, Jovicich J. Length matters: Improved high field EEG–fMRI recordings using shorter EEG cables. *Journal of Neuroscience Methods* **269** (2016) 74–87. doi:10.1016/j.jneumeth.2016.05.014.

Allen PJ, Polizzi G, Krakow K, Fish DR, Lemieux L. Identification of EEG events in the MR scanner: the problem of pulse artifact and a method for its subtraction. *NeuroImage* **8** (1998) 229–239. doi:10.1006/nimg.1998.0361.

Goldman RI, Stern JM, Engel J, Cohen MS. Acquiring simultaneous EEG and functional MRI. *Clinical Neurophysiology: Official Journal of the International Federation of Clinical Neurophysiology* **111** (2000) 1974–1980.

Jorge J, Grouiller F, Ipek Stoermer R, Michel CM, Figueiredo P, et al. Simultaneous EEG–fMRI at ultra-high field: Artifact prevention and safety assessment. *NeuroImage* **105** (2015a) 132–144. doi:10.1016/j.neuroimage.2014.10.055.

Mullinger KJ, Chowdhury MEH, Bowtell R. Investigating the effect of modifying the EEG cap lead configuration on the gradient artifact in simultaneous EEG-fMRI. *Frontiers in Neuroscience* **8** (2014) 226. doi:10.3389/fnins.2014.00226.

Poulsen C, Wakeman DG, Atefi SR, Luu P, Konyn A, Bonmassar G. Polymer thick film technology for improved simultaneous dEEG/MRI recording: Safety and MRI data quality. *Magnetic Resonance in Medicine* **77** (2017) 895–903. doi:10.1002/mrm.26116.

Chowdhury MEH, Khandakar A, Mullinger K, Hossain B, Al-Emadi N, Antunes A, et al. Reference Layer Artefact Subtraction (RLAS): Electromagnetic Simulations. *Ieee Access* **7** (2019) 17882–17895. doi:10.1109/ACCESS.2019.2892766. WOS:000459164900001.

Mandelkow H, Halder P, Boesiger P, Brandeis D. Synchronization facilitates removal of MRI artefacts from concurrent EEG recordings and increases usable bandwidth. *NeuroImage* **32** (2006) 1120–1126. doi:10.1016/j.neuroimage.2006.04.231.

Mullinger KJ, Morgan PS, Bowtell RW. Improved artifact correction for combined electroencephalography/functional MRI by means of synchronization and use of vectorcardiogram recordings. *Journal of Magnetic Resonance Imaging* **27** (2008a) 607–616. doi:10.1002/jmri.21277.

Gebhardt H, Blecker CR, Bischoff M, Morgen K, Oschmann P, Vaitl D, et al. Synchronized measurement of simultaneous EEG-fMRI: a simulation study. *Clinical Neurophysiology: Official Journal of the International Federation of Clinical Neurophysiology* **119** (2008) 2703–2711. doi:10.1016/j.clinph.2008.09.018.

Solana AB, Hernández-Tamames JA, Manzanedo E, García-Álvarez R, Zelaya FO, del Pozo F. Gradient induced artifacts in simultaneous EEG-fMRI: Effect of synchronization on spiral and EPI k-space trajectories. *Magnetic Resonance Imaging* **32** (2014) 684–692. doi:10.1016/j.mri.2014.03.008.

Goncalves SI, Pouwels PJW, Kuijer JPA, Heethaar RM, de Munck JC. Artifact removal in co-registered EEG/fMRI by selective average subtraction. *Clinical Neurophysiology: Official Journal of the International Federation of Clinical Neurophysiology* **118** (2007a) 2437–2450. doi:10.1016/j.clinph.2007.08.017.

Goncalves SI, Pouwels PJW, Kuijer JPA, Heethaar RM, de Munck JC. Correction for desynchronization of EEG and fMRI clocks through data interpolation optimizes artifact reduction. *2007 Annual International Conference of the Ieee Engineering in Medicine and Biology Society, Vols 1-16* (New York: Ieee) (2007b), 1590–1594. WOS:000253467001083.

Koskinen M, Vartiainen N. Removal of imaging artifacts in EEG during simultaneous EEG/fMRI recording: reconstruction of a high-precision artifact template. *NeuroImage* **46** (2009) 160–167. doi:10.1016/j.neuroimage.2009.01.061.

Mandelkow H, Brandeis D, Boesiger P. Good practices in EEG-MRI: The utility of retrospective synchronization and PCA for the removal of MRI gradient artefacts. *Neuroimage* **49** (2010) 2287–2303. doi:10.1016/j.neuroimage.2009.10.050. WOS:000273626400034.

Tan A, Tu Y, Fu Z, Huang G, Hung YS, Zhang Z. A Least Across-segment Variance (LASV) Method for the Correction of EEG-fMRI Desynchronization. *2017 8th International Ieee/Embs Conference on Neural Engineering (ner)* (New York: Ieee) (2017), 5–8. WOS:000428143200002.

Allen PJ, Josephs O, Turner R. A method for removing imaging artifact from continuous EEG recorded during functional MRI. *NeuroImage* **12** (2000) 230–239. doi:10.1006/nimg.2000.0599.

Garreffa G, Carnì M, Gualniera G, Ricci GB, Bozzao L, De Carli D, et al. Real-time MR artifacts filtering during continuous EEG/fMRI acquisition. *Magnetic Resonance Imaging* **21** (2003) 1175–1189.

Gualniera G, Garreffa G, Morasso P, Carni M, Granozio G, Repetti A, et al. A method for real-time artifact filtering during simultaneous EEG/fMRI acquisition: preliminary results. *Neurocomputing* **58** (2004) 1171–1179. doi:10.1016/j.neucom.2004.01.182. WOS:000222245900168.

Moosmann M, Schönfelder VH, Specht K, Scheeringa R, Nordby H, Hugdahl K. Realignment parameter-informed artefact correction for simultaneous EEG-fMRI recordings. *NeuroImage* **45** (2009) 1144–1150. doi:10.1016/j.neuroimage.2009.01.024.

Sun L, Rieger J, Hinrichs H. Maximum noise fraction (MNF) transformation to remove ballistocardiographic artifacts in EEG signals recorded during fMRI scanning. *NeuroImage* **46** (2009) 144–153. doi:10.1016/j.neuroimage.2009.01.059.

Zhang S, Hennig J, LeVan P. Direct modelling of gradient artifacts for EEG-fMRI denoising and motion tracking. *Journal of Neural Engineering* **16** (2019) 056010. doi:10.1088/1741-2552/ab2b21. WOS:000479107700001.

Wan X, Iwata K, Riera J, Kitamura M, Kawashima R. Artifact reduction for simultaneous EEG/fMRI recording: adaptive FIR reduction of imaging artifacts. *Clinical Neurophysiology: Official Journal of the International Federation of Clinical Neurophysiology* **117** (2006a) 681–692. doi:10.1016/j.clinph.2005.07.025.

Freyer F, Becker R, Anami K, Curio G, Villringer A, Ritter P. Ultrahigh-frequency EEG during fMRI: pushing the limits of imaging-artifact correction. *NeuroImage* **48** (2009) 94–108. doi:10.1016/j.neuroimage.2009.06.022.

Sartori E, Formaggio E, Storti SF, Bertoldo A, Manganotti P, Fiaschi A, et al. Gradient Artifact Removal in Co-registration EEG/fMRI. Dossel O, Schlegel WC, editors, *World Congress on Medical Physics and Biomedical Engineering, Vol 25, Pt 4: Image Processing, Biosignal Processing, Modelling and Simulation, Biomechanics* (New York: Springer), vol. 25 (2010), 1143–1146. WOS:000300975300304.

Huang CH, Ju MS, Lin CCK. A robust algorithm for removing artifacts in EEG recorded during FMRI/EEG study. *Computers in Biology and Medicine* **42** (2012) 458–467. doi:10.1016/j.compbiomed.2011.12.014.

Ferreira JL, Aarts RM, Cluitmans PJM. Optimized Moving-Average Filtering for Gradient Artefact Correction During Simultaneous EEG-fMRI. *5th Issnip-Ieee Biosignals and Biorobotics Conference (2014): Biosignals and Robotics for Better and Safer Living* (New York: Ieee) (2014), 1–6. WOS:000345908100001.

LeVan P, Zhang S, Knowles B, Zaitsev M, Hennig J. EEG-fMRI Gradient Artifact Correction by Multiple Motion-Related Templates. *IEEE Transactions on Biomedical Engineering* **63** (2016) 2647–2653. doi:10.1109/TBME.2016.2593726.

de Munck JC, van Houdt PJ, Gonçalves SI, van Wegen E, Ossenblok PPW. Novel artefact removal algorithms for co-registered EEG/fMRI based on selective averaging and subtraction. *NeuroImage* **64** (2013) 407–415. doi:10.1016/j.neuroimage.2012.09.022.

Ryali S, Glover GH, Chang C, Menon V. Development, validation, and comparison of ICA-based gradient artifact reduction algorithms for simultaneous EEG-spiral in/out and echo-planar fMRI recordings. *NeuroImage* **48** (2009) 348–361. doi:10.1016/j.neuroimage.2009.06.072.

Acharjee PP, Phlypo R, Wu L, Calhoun VD, Adali T. Gradient Artifact Removal in Concurrently Acquired Eeg Data Using Independent Vector Analysis. *2014 Ieee International Conference on Acoustics, Speech and Signal Processing (icassp)* (New York: Ieee) (2014). WOS:000343655305179.

Acharjee PP, Phlypo R, Wu L, Calhoun VD, Adali T. Independent Vector Analysis for Gradient Artifact Removal in Concurrent EEG-fMRI Data. *IEEE transactions on bio-medical engineering* **62** (2015) 1750–1758. doi:10.1109/TBME.2015.2403298.

Negishi M, Abildgaard M, Nixon T, Constable RT. Removal of time-varying gradient artifacts from EEG data acquired during continuous fMRI. *Clinical Neurophysiology: Official Journal of the International Federation of Clinical Neurophysiology* **115** (2004) 2181–2192. doi:10.1016/j.clinph.2004.04.005.

Niazy RK, Beckmann CF, Iannetti GD, Brady JM, Smith SM. Removal of FMRI environment artifacts from EEG data using optimal basis sets. *NeuroImage* **28** (2005) 720–737. doi:10.1016/j.neuroimage.2005.06.067.

Liu Z, de Zwart JA, van Gelderen P, Kuo LW, Duyn JH. Statistical feature extraction for artifact removal from concurrent fMRI-EEG recordings. *NeuroImage* **59** (2012) 2073–2087. doi:10.1016/j.neuroimage.2011.10.042.

Li J, Chen Y, Taya F, Lim J, Wong K, Sun Y, et al. A unified canonical correlation analysis-based framework for removing gradient artifact in concurrent EEG/fMRI recording and motion artifact in walking recording from EEG signal. *Medical & Biological Engineering & Computing* **55** (2017) 1669–1681. doi:10.1007/s11517-017-1620-3.

Jing M, Sanei S. Scanner artifact removal in simultaneous EEG-fMRI for epileptic seizure prediction. Tang YY, Wang SP, Lorette G, Yeung DS, Yan H, editors, *18th International Conference on Pattern Recognition, Vol 3, Proceedings* (Los Alamitos: Ieee Computer Soc) (2006), 722–+. WOS:000240705600173.

Hoffmann A, Jager L, Werhahn KJ, Jaschke M, Noachtar S, Reiser M. Electroencephalography during functional echo-planar imaging: Detection of epileptic spikes using post-processing methods. *Magnetic Resonance in Medicine* **44** (2000) 791–798. doi:10.1002/1522-2594(200011)44:5⟨791::AID-MRM17⟩3.0.CO;2-2. WOS:000165163600017.

Shaw SB. Real-Time Filtering of Gradient Artifacts from Simultaneous EEG-fMRI Data. *2017 International Workshop on Pattern Recognition in Neuroimaging (prni)* (New York: IEEE) (2017). WOS:000426948900016.

Frigo G, Narduzzi C. *EEG Gradient Artifact Removal by Compressive Sensing and Taylor-Fourier Transform* (New York: Ieee) (2014). WOS:000346747000059.

Xu P, Chen H, Liu Z, Yao D. A new method based on sparse component decomposition to remove MRI artifacts in the continuous EEG recordings. *2005 27th Annual International Conference of the IEEE Engineering in Medicine and Biology Society, Vols 1-7* (New York: Ieee) (2005), 2006–2008. WOS:000238998401248.

Ertl M, Kirsch V, Leicht G, Karch S, Olbrich S, Reiser M, et al. Avoiding the ballistocardiogram (BCG) artifact of EEG data acquired simultaneously with fMRI by pulse-triggered presentation of stimuli. *Journal of Neuroscience Methods* **186** (2010) 231–241. doi:10.1016/j.jneumeth.2009.11.009.

Dyrholm M, Goldman R, Sajda P, Brown TR. Removal of BCG artifacts using a non-Kirchhoffian overcomplete representation. *IEEE transactions on bio-medical engineering* **56** (2009) 200–204. doi:10.1109/TBME.2008.2005952.

Klovatch-Podlipsky I, Gazit T, Fahoum F, Tsirelson B, Kipervasser S, Kremer U, et al. Dual array EEG-fMRI: An approach for motion artifact suppression in EEG recorded simultaneously with fMRI. *NeuroImage* **142** (2016) 674–686. doi:10.1016/j.neuroimage.2016.07.014.

Cohen N, Tsizin E, Fried I, Fahoum F, Hendler T, Gazit T, et al. Conductive gel bridge sensor for motion tracking in simultaneous EEG-fMRI recordings. *Epilepsy Research* **149** (2019) 117–122. doi:10.1016/j.eplepsyres.2018.12.008. WOS:000456751500019.

Xia H, Ruan D, Cohen MS. BCG Artifact Removal for Reconstructing Full-scalp EEG inside the MR Scanner. *2013 3rd International Workshop on Pattern Recognition in Neuroimaging (prni 2013)* (New York: Ieee) (2013a), 178–181. WOS:000333958600044.

Xia H, Ruan D, Cohen MS. Coupled Basis Learning and Regularized Reconstruction for Bcg Artifact Removal in Simultaneous Eeg-Fmri Studies. *2013 Ieee 10th International Symposium on Biomedical Imaging (isbi)* (New York: Ieee) (2013b), 986–989. WOS:000326900100247.

Xia H, Ruan D, Cohen MS. Removing ballistocardiogram (BCG) artifact from full-scalp EEG acquired inside the MR scanner with Orthogonal Matching Pursuit (OMP). *Frontiers in Neuroscience* **8** (2014a). doi:10.3389/fnins.2014.00218.

Xia H, Ruan D, Cohen MS. Separation and reconstruction of BCG and EEG signals during continuous EEG and fMRI recordings. *Frontiers in Neuroscience* **8** (2014b). doi:10.3389/fnins.2014.00163.

Jorge J, Grouiller F, Gruetter R, van der Zwaag W, Figueiredo P. Towards high-quality simultaneous EEG-fMRI at 7T: Detection and reduction of EEG artifacts due to head motion. *NeuroImage* **120** (2015b) 143–153. doi:10.1016/j.neuroimage.2015.07.020.

Chowdhury MEH, Mullinger KJ, Glover P, Bowtell R. Reference layer artefact subtraction (RLAS): A novel method of minimizing EEG artefacts during simultaneous fMRI. *NeuroImage* **84** (2014) 307–319. doi:10.1016/j.neuroimage.2013.08.039.

Luo Q, Huang X, Glover GH. Ballistocardiogram artifact removal with a reference layer and standard EEG cap. *Journal of Neuroscience Methods* **233** (2014) 137–149. doi:10.1016/j.jneumeth.2014.06.021.

Steyrl D, Patz F, Krausz G, Edlinger G, Mueller-Putz GR. Reduction of EEG Artifacts in Simultaneous EEG-fMRI: Reference Layer Adaptive Filtering (RLAF). *2015 37th Annual International Conference of the Ieee Engineering in Medicine and Biology Society (embc)* (New York: Ieee) (2015), 3803–3806. WOS:000371717204022.

Steyrl D, Krausz G, Koschutnig K, Edlinger G, Müller-Putz GR. Reference layer adaptive filtering (RLAF) for EEG artifact reduction in simultaneous EEG-fMRI. *Journal of Neural Engineering* **14** (2017) 026003. doi:10.1088/1741-2552/14/2/026003.

Steyrl D, Krausz G, Koschutnig K, Edlinger G, Müller-Putz GR. Online Reduction of Artifacts in EEG of Simultaneous EEG-fMRI Using Reference Layer Adaptive Filtering (RLAF). *Brain Topography* **31** (2018) 129–149. doi:10.1007/s10548-017-0606-7.

Bonmassar G, Purdon PL, Jääskeläinen IP, Chiappa K, Solo V, Brown EN, et al. Motion and ballistocardiogram artifact removal for interleaved recording of EEG and EPs during MRI. *NeuroImage* **16** (2002) 1127–1141.

Iannotti GR, Pittau F, Michel CM, Vulliemoz S, Grouiller F. Pulse artifact detection in simultaneous EEG-fMRI recording based on EEG map topography. *Brain Topography* **28** (2015) 21–32. doi:10.1007/s10548-014-0409-z.

Masterton RAJ, Abbott DF, Fleming SW, Jackson GD. Measurement and reduction of motion and ballistocardiogram artefacts from simultaneous EEG and fMRI recordings. *NeuroImage* **37** (2007) 202–211. doi:10.1016/j.neuroimage.2007.02.060.

Abbott DF, Masterton RAJ, Archer JS, Fleming SW, Warren AEL, Jackson GD. Constructing Carbon Fiber Motion-Detection Loops for Simultaneous EEG-fMRI. *Frontiers in Neurology* **5** (2014) 260. doi:10.3389/fneur.2014.00260.

van der Meer JN, Pampel A, Van Someren EJW, Ramautar JR, van der Werf YD, Gomez-Herrero G, et al. Carbon-wire loop based artifact correction outperforms post-processing EEG/fMRI corrections—A validation of a real-time simultaneous EEG/fMRI correction method. *NeuroImage* **125** (2016a) 880–894. doi:10.1016/j.neuroimage.2015.10.064.

van der Meer JN, Pampel A, van Someren E, Ramautar J, van der Werf Y, Gomez-Herrero G, et al. "Eyes Open – Eyes Closed" EEG/fMRI data set including dedicated "Carbon Wire Loop" motion detection channels. *Data in Brief* **7** (2016b) 990–994. doi:10.1016/j.dib.2016.03.001.

Kim KH, Yoon HW, Park HW. Improved ballistocardiac artifact removal from the electroencephalogram recorded in fMRI. *Journal of Neuroscience Methods* **135** (2004) 193–203. doi:10.1016/j.jneumeth.2003.12.016.

Kustra AJL, Fernandes JM, Cunha JPS. EEG-fMRI Ballistocardiogram Removal: A New Non-linear Dynamic Time Warping Approach. Katashev A, Dekhtyar Y, Spigulis J, editors, *14th Nordic-Baltic Conference on Biomedical Engineering and Medical Physics* (New York: Springer), vol. 20 (2008), 278–281. WOS:000260668000075.

Ellingson ML, Liebenthal E, Spanaki MV, Prieto TE, Binder JR, Ropella KM. Reduction of ballistocardiogram artifact in the simultaneous acquisition of auditory event-related potentials and functional magnetic resonance images. *Second Joint Embs-Bmes Conference 2002, Vols 1-3, Conference Proceedings: Bioengineering - Integrative Methodologies, New Technologies* (New York: Ieee) (2002), 159–160. WOS:000180194800075.

Ellingson ML, Liebenthal E, Spanaki MV, Prieto TE, Binder JR, Ropella KM. Ballistocardiogram artifact reduction in the simultaneous acquisition of auditory ERPS and fMRI. *NeuroImage* **22** (2004) 1534–1542. doi:10.1016/j.neuroimage.2004.03.033.

Oh SS, Chung JY, Yoon HW, Park H. An accurate heart beat detection method in the EKG recorded in fMRI system. *2007 Annual International Conference of the Ieee Engineering in Medicine and Biology Society, Vols 1-16* (New York: Ieee) (2007), 656–+. WOS:000253467000167.

Oh SS, Han Y, Lee J, Yun SD, Kang JK, Lee EM, et al. A pulse artifact removal method considering artifact variations in the simultaneous recording of EEG and fMRI. *Neuroscience Research* **81-82** (2014) 42–50. doi:10.1016/j.neures.2014.01.008.

Wen X, Kang M, Yao L, Zhao X. Real-time ballistocardiographic artifact reduction using the k-teager energy operator detector and multi-channel referenced adaptive noise cancelling. *International Journal of Imaging Systems and Technology* **26** (2016) 209–215. doi:10.1002/ima.22178. WOS:000383475300005.

Wong CK, Luo Q, Zotev V, Phillips R, Chan KWC, Bodurka J. Automatic cardiac cycle determination directly from EEG-fMRI data by multi-scale peak detection method. *Journal of Neuroscience Methods* (2018). doi:10.1016/j.jneumeth.2018.03.017.

Vincent JL, Larson-Prior LJ, Zempel JM, Snyder AZ. Moving GLM ballistocardiogram artifact reduction for EEG acquired simultaneously with fMRI. *Clinical Neurophysiology: Official Journal of the International Federation of Clinical Neurophysiology* **118** (2007) 981–998. doi:10.1016/j.clinph.2006.12.017.

Bénar CG, Aghakhani Y, Wang Y, Izenberg A, Al-Asmi A, Dubeau F, et al. Quality of EEG in simultaneous EEG-fMRI for epilepsy. *Clinical Neurophysiology* **114** (2003) 569–580. doi:10.1016/S1388-2457(02)00383-8.

Briselli E, Garreffa G, Bianchi L, Bianciardi M, Macaluso E, Abbafati M, et al. An independent component analysis-based approach on ballistocardiogram artifact removing. *Magnetic Resonance Imaging* **24** (2006) 393–400. doi:10.1016/j.mri.2006.01.008.

Koskinen M, Vartiainen N. Removal of ballistocardiogram artifact from EEG data acquired in the MRI scanner: selection of ICA components. *2008 30th Annual International Conference of the Ieee Engineering in Medicine and Biology Society, Vols 1-8* (New York: Ieee) (2008), 5220–+. WOS:000262404503107.

Ferdowsi S, Sanei S, Nottage J, O'Daly O, Abolghasemi V. A Hybrid Ica-Hermite Transform for Removal of Ballistocardiogram from Eeg. *2012 Proceedings of the 20th European Signal Processing Conference (eusipco)* (Los Alamitos: Ieee Computer Soc) (2012a), 484–488. WOS:000310623800098.

Silva de Souza AC, Rodrigues GF, Callan D, Yehia HC. Analysis of the Ballistocardiographic Artifact Removal in Simultaneous EEG-fMRI Recording Using Independent Component Analysis and Coherence Function. Herencsar N, Molnar K, editors, *2013 36th International Conference on Telecommunications and Signal Processing (TSP)* (New York: IEEE) (2013), 552–556. WOS:000333968000111.

Abbasi O, Dammers J, Arrubla J, Warbrick T, Butz M, Neuner I, et al. Time-frequency analysis of resting state and evoked EEG data recorded at higher magnetic fields up to 9.4 T. *Journal of Neuroscience Methods* **255** (2015) 1–11. doi:10.1016/j.jneumeth.2015.07.011.

Abreu R, Leite M, Jorge J, Grouiller F, van der Zwaag W, Leal A, et al. Ballistocardiogram artifact correction taking into account physiological signal preservation in simultaneous EEG-fMRI. *NeuroImage* **135** (2016) 45–63. doi:10.1016/j.neuroimage.2016.03.034.

Piorecky M, Koudelka V, Strobl J, Brunovsky M, Krajca V. Artifacts in Simultaneous hdEEG/fMRI Imaging: A Nonlinear Dimensionality Reduction Approach. *Sensors (Basel, Switzerland)* **19** (2019). doi:10.3390/s19204454.

Rasheed T, In MH, Lee YK, Lee S, Lee SY, Kim TS. Constrained ICA based ballistocardiogram and electro-oculogram artifacts removal from visual evoked potential EEG signals measured inside MRI. King I, Wang J, Chan L, Wang DL, editors, *Neural Information Processing, Pt 1, Proceedings* (Berlin: Springer-Verlag Berlin), vol. 4232 (2006), 1088–1097. WOS:000241790100121.

Rasheed T, Lee YK, Lee SY, Kim TS. Attenuation of artifacts in EEG signals measured inside an MRI scanner using constrained independent component analysis. *Physiological Measurement* **30** (2009) 387–404. doi:10.1088/0967-3334/30/4/004.

Leclercq Y, Balteau E, Dang-Vu T, Schabus M, Luxen A, Maquet P, et al. Rejection of pulse related artefact (PRA) from continuous electroencephalographic (EEG) time series recorded during functional magnetic resonance imaging (fMRI) using constraint independent component analysis (cICA). *NeuroImage* **44** (2009) 679–691. doi:10.1016/j.neuroimage.2008.10.017.

Wang K, Li W, Dong L, Zou L, Wang C. Clustering-Constrained ICA for Ballistocardiogram Artifacts Removal in Simultaneous EEG-fMRI. *Frontiers in Neuroscience* **12** (2018). doi:10.3389/fnins.2018.00059.

Javed E, Faye I, Malik AS, Abdullah JM. A Hybrid Method to Improve the Reduction of Ballistocardiogram Artifact from EEG Data. Loo CK, Yap KS, Wong KW, Teoh A, Huang K, editors, *Neural Information Processing (iconip 2014), Pt Ii* (Cham: Springer International Publishing Ag), vol. 8835 (2014a), 186–193. WOS:000432982300023.

Javed E, Faye I, Malik AS, Abdullah JM. Reference-free Reduction of Ballistocardiogram Artifact from EEG Data Using EMD-PCA. *2014 5th International Conference on Intelligent and Advanced Systems (icias 2014)* (New York: Ieee) (2014b). WOS:000345738000070.

Javed E, Faye I, Malik AS, Abdullah JM. *An EMD based Method for Reduction of Ballistocardiogram Artifact from EEG Studies of Evoked Potentials* (Amsterdam: Elsevier Science Bv) (2015). WOS:000380483600208.

Javed E, Faye I, Malik AS, Abdullah JM. Removal of BCG artefact from concurrent fMRI-EEG recordings based on EMD and PCA. *Journal of Neuroscience Methods* **291** (2017) 150–165. doi:10.1016/j.jneumeth.2017.08.020.

Wu X, Wu T, Zhan Z, Yao L, Wen X. A real-time method to reduce ballistocardiogram artifacts from EEG during fMRI based on optimal basis sets (OBS). *Computer Methods and Programs in Biomedicine* **127**

(2016) 114–125. doi:10.1016/j.cmpb.2016.01.018.

Marino M, Liu Q, Koudelka V, Porcaro C, Hlinka J, Wenderoth N, et al. Adaptive optimal basis set for BCG artifact removal in simultaneous EEG-fMRI. *Scientific Reports* **8** (2018) 8902. doi:10.1038/s41598-018-27187-6.

Assecondi S, Van Hese P, Hallez H, D'Asseler Y, Lemahieu I, Bianchi AM, et al. *Ballistocardiographic artifact removal from simultaneous EEG/FMRI recording by means of canonical correlation analysis* (Setubal: Insticc-Inst Syst Technologies Information Control & Communication) (2008). WOS:000256983100005.

Assecondi S, Hallez H, Staelens S, Bianchi AM, Huiskamp GM, Lemahieu I. Removal of the ballistocardiographic artifact from EEG-fMRI data: a canonical correlation approach. *Physics in Medicine and Biology* **54** (2009) 1673–1689. doi:10.1088/0031-9155/54/6/018.

Kosma A, Nazarpour K, Sanei S. *Removal of Ballistocardiogram Artifact from Electroencephalograms Exploiting Heart Rate Variability* (New York: Ieee) (2009). WOS:000276494500046.

Ferdowsi S, Abolghasemi V, Sanei S. Blind Separation of Ballistocardiogram from Eeg Via Short-and-Long-Term Linear Prediction Filtering. Santamaria I, ArenasGarcia J, CampsValls G, Erdogmus D, PerezCruz F, Larsen J, editors, *2012 Ieee International Workshop on Machine Learning for Signal Processing (mlsp)* (New York: Ieee) (2012b). WOS:000311966000070.

Ferdowsi S, Sanei S, Abolghasemi V, Nottage J, O'Daly O. Removing ballistocardiogram artifact from EEG using short- and long-term linear predictor. *IEEE transactions on bio-medical engineering* **60** (2013) 1900–1911. doi:10.1109/TBME.2013.2244888.

Siniatchkin M, Moeller F, Jacobs J, Stephani U, Boor R, Wolff S, et al. Spatial filters and automated spike detection based on brain topographies improve sensitivity of EEG-fMRI studies in focal epilepsy. *NeuroImage* **37** (2007) 834–843. doi:10.1016/j.neuroimage.2007.05.049.

Ghaderi F, Nazarpour K, McWhirter JG, Sanei S. Removal of Ballistocardiogram Artifacts Using the Cyclostationary Source Extraction Method. *Ieee Transactions on Biomedical Engineering* **57** (2010) 2667–2676. doi:10.1109/TBME.2010.2060334. WOS:000283439900004.

Krishnaswamy P, Bonmassar G, Purdon PL, Brown EN. Reference-Free Harmonic Regression Technique to Remove EEG-fMRI Ballistocardiogram Artifacts. *2013 35th Annual International Conference of the Ieee Engineering in Medicine and Biology Society (embc)* (New York: Ieee) (2013), 5426–5429. WOS:000341702105207.

Krishnaswamy P, Bonmassar G, Poulsen C, Pierce ET, Purdon PL, Brown EN. Reference-free removal of EEG-fMRI ballistocardiogram artifacts with harmonic regression. *NeuroImage* **128** (2016) 398–412. doi:10.1016/j.neuroimage.2015.06.088.

Wan X, Iwata K, Riera J, Ozaki T, Kitamura M, Kawashima R. Artifact reduction for EEG/fMRI recording: nonlinear reduction of ballistocardiogram artifacts. *Clinical Neurophysiology: Official Journal of the International Federation of Clinical Neurophysiology* **117** (2006b) 668–680. doi:10.1016/j.clinph.2005.12.015.

Abolghasemi V, Ferdowsi S. EEG-fMRI: Dictionary learning for removal of ballistocardiogram artifact from EEG. *Biomedical Signal Processing and Control* **18** (2015) 186–194. doi:10.1016/j.bspc.2015.01.001. WOS:000354150400024.

In MH, Lee SY, Park TS, Kim TS, Cho MH, Ahn YB. Ballistocardiogram artifact removal from EEG signals using adaptive filtering of EOG signals. *Physiological Measurement* **27** (2006) 1227–1240. doi:10.1088/0967-3334/27/11/014. WOS:000241517800014.

Sameni R, Shamsollahi MB, Jutten C. Model-based Bayesian filtering of cardiac contaminants from biomedical recordings. *Physiological Measurement* **29** (2008) 595–613. doi:10.1088/0967-3334/29/5/

006. WOS:000256352100006.

Mahadevan A, Mugler DH, Acharya S. Adaptive Filtering of Ballistocardiogram Artifact from EEG signals Using the Dilated Discrete Hermite Transform. *2008 30th Annual International Conference of the Ieee Engineering in Medicine and Biology Society, Vols 1-8* (New York: Ieee) (2008a), 2630–+. WOS:000262404501260.

Mahadevan A, Acharya S, Sheffer DB, Mugler DH. Ballistocardiogram Artifact Removal in EEG-fMRI Signals Using Discrete Hermite Transforms. *Ieee Journal of Selected Topics in Signal Processing* **2** (2008b) 839–853. doi:10.1109/JSTSP.2008.2008367. WOS:000265495700004.

Bonmassar G, Anami K, Ives J, Belliveau JW. Visual evoked potential (VEP) measured by simultaneous 64-channel EEG and 3T fMRI. *Neuroreport* **10** (1999) 1893–1897.

Ruggieri A, Vaudano AE, Benuzzi F, Serafini M, Gessaroli G, Farinelli V, et al. Mapping (and modeling) physiological movements during EEG–fMRI recordings: The added value of the video acquired simultaneously. *Journal of Neuroscience Methods* **239** (2015) 223–237. doi:10.1016/j.jneumeth.2014.10.005.

LeVan P, Maclaren J, Herbst M, Sostheim R, Zaitsev M, Hennig J. Ballistocardiographic artifact removal from simultaneous EEG-fMRI using an optical motion-tracking system. *NeuroImage* **75** (2013) 1–11. doi:10.1016/j.neuroimage.2013.02.039.

Kruggel F, Wiggins CJ, Herrmann CS, von Cramon DY. Recording of the event-related potentials during functional MRI at 3.0 Tesla field strength. *Magnetic Resonance in Medicine* **44** (2000) 277–282. doi:10.1002/1522-2594(200008)44:2⟨277::AID-MRM15⟩3.0.CO;2-X. WOS:000088545600015.

Mullinger KJ, Brookes M, Stevenson C, Morgan P, Bowtell R. Exploring the feasibility of simultaneous electroencephalography/functional magnetic resonance imaging at 7 T. *Magnetic Resonance Imaging* **26** (2008b) 968–977. doi:10.1016/j.mri.2008.02.014. WOS:000258806500015.

Rothluebbers S, Relvas V, Leal A, Figueiredo P. Reduction of EEG artefacts induced by vibration in the MR-environment. *2013 35th Annual International Conference of the Ieee Engineering in Medicine and Biology Society (embc)* (New York: Ieee) (2013), 2092–2095. WOS:000341702102141.

Kim HC, Yoo SS, Lee JH. Recursive approach of EEG-segment-based principal component analysis substantially reduces cryogenic pump artifacts in simultaneous EEG–fMRI data. *NeuroImage* **104** (2015) 437–451. doi:10.1016/j.neuroimage.2014.09.049.

Mayeli A, Zotev V, Refai H, Bodurka J. An Automatic ICA-Based Method for Removing Artifacts from EEG Data Acquired during fMRI in Real Time. *2015 41st Annual Northeast Biomedical Engineering Conference (nebec)* (New York: Ieee) (2015). WOS:000370977700019.

Mayeli A, Zotev V, Refai H, Bodurka J. Real-time EEG artifact correction during fMRI using ICA. *Journal of Neuroscience Methods* **274** (2016) 27–37. doi:10.1016/j.jneumeth.2016.09.012.

Porcaro C, Ostwald D, Bagshaw AP. Functional source separation improves the quality of single trial visual evoked potentials recorded during concurrent EEG-fMRI. *NeuroImage* **50** (2010) 112–123. doi:10.1016/j.neuroimage.2009.12.002.

Brookes MJ, Mullinger KJ, Stevenson CM, Morris PG, Bowtell R. Simultaneous EEG source localisation and artifact rejection during concurrent fMRI by means of spatial filtering. *NeuroImage* **40** (2008) 1090–1104. doi:10.1016/j.neuroimage.2007.12.030.

Brookes MJ, Vrba J, Mullinger KJ, Geirsdottir GB, Yan WX, Stevenson CM, et al. Source localisation in concurrent EEG/fMRI: Applications at 7T. *Neuroimage* **45** (2009) 440–452. doi:10.1016/j.neuroimage.2008.10.047. WOS:000263863000023.

Warach S, Ives JR, Schlaug G, Patel MR, Darby DG, Thangaraj V, et al. EEG-triggered echo-planar functional MRI in epilepsy. *Neurology* **47** (1996) 89–93. doi:10.1212/WNL.47.1.89.

Onojima T, Kitajo K, Mizuhara H. Ongoing slow oscillatory phase modulates speech intelligibility in cooperation with motor cortical activity. *Plos One* **12** (2017) e0183146. doi:10.1371/journal.pone.0183146. WOS:000407431800059.

Anwar AR, Muthalib M, Perrey S, Galka A, Granert O, Wolff S, et al. Effective Connectivity of Cortical Sensorimotor Networks During Finger Movement Tasks: A Simultaneous fNIRS, fMRI, EEG Study. *Brain Topography* **29** (2016) 645–660. doi:10.1007/s10548-016-0507-1. WOS:000382009200001.

Baenninger A, Diaz Hernandez L, Rieger K, Ford JM, Kottlow M, Koenig T. Inefficient Preparatory fMRI-BOLD Network Activations Predict Working Memory Dysfunctions in Patients with Schizophrenia. *Frontiers in Psychiatry* **7** (2016) 29. doi:10.3389/fpsyt.2016.00029. WOS:000372368100002.

Wirsich J, Ridley B, Besson P, Jirsa V, Bénar C, Ranjeva JP, et al. Complementary contributions of concurrent EEG and fMRI connectivity for predicting structural connectivity. *NeuroImage* **161** (2017) 251–260. doi:10.1016/j.neuroimage.2017.08.055.
